# GFICLEE: ultrafast tree-based phylogenetic profile method inferring gene function at the genomic-wide level

**DOI:** 10.1186/s12864-021-08070-7

**Published:** 2021-10-29

**Authors:** Yang Fang, Menglong Li, Xufeng Li, Yi Yang

**Affiliations:** 1 Key Laboratory of Bio-Resources and Eco-Environment of Ministry of Education, College of Life Sciences, Sichuan University, Chengdu, People’s Republic of China; 2 College of Chemistry, Sichuan University, Chengdu, People’s Republic of China

**Keywords:** Phylogenetic profile, Tree-base method, Gene function, Genome-wide

## Abstract

**Background:**

Phylogenetic profiling is widely used to predict novel members of large protein complexes and biological pathways. Although methods combined with phylogenetic trees have significantly improved prediction accuracy, computational efficiency is still an issue that limits its genome-wise application.

**Results:**

Here we introduce a new tree-based phylogenetic profiling algorithm named GFICLEE, which infers common single and continuous loss (SCL) events in the evolutionary patterns. We validated our algorithm with human pathways from three databases and compared the computational efficiency with current tree-based with 10 different scales genome dataset. Our algorithm has a better predictive performance with high computational efficiency.

**Conclusions:**

The GFICLEE is a new method to infers genome-wide gene function. The accuracy and computational efficiency of GFICLEE make it possible to explore gene functions at the genome-wide level on a personal computer.

**Supplementary Information:**

The online version contains supplementary material available at 10.1186/s12864-021-08070-7.

## Background

Phylogenetic profiles inferring the functions of protein-coding genes based on shared binary patterns of homology gain and loss. For the first time, the phylogenetic profile is used to predict gene function that using the binary phylogenetic profile with the presence and the absence of homologies of a reference genome across organisms [1]. Phylogenetic profile plays a critical role in exploring gene functions. Such as, phylogenetic profiles have been used to infer the function network [2], gene fusions [3], protein-protein interactions [4–6], and gene function explored [7–10]. Over the past decade, the rapid development of sequencing technologies, especially the development of high-throughput sequencing technologies, has led to a linear decline in sequencing costs, enabling phylogenetic techniques to be applied at the genome-wide level of eukaryotes. For example, our previous work introduced PrePhyloPro, web-based software that is based on the phylogenetic profile for accurately predicting proteome-wide linkages [5]. There are also other applications of the phylogenetic profile in *Saccharomyces cerevisiae* (*S. cerevisiae*) [3], *Drosophila melanogaster* (*D. melanogaster*) [11], and *Caenorhabditis elegans* (*C. elegans*) [12]. The binary phylogenetic profile that can be used to explore gene function. However, there has a basic challenge is how to identify co-evolve genes with the same function by similar patterns of presence and absence. The measure methods always used to compare phylogenetic profiles are Hamming distance [1, 13], Jaccard similarity [14, 15] and Pearson correlation [16]. On other hand, in non-binary phylogenetic profiles, Mutual information is often used to measure similarities between phylogenetic profile vectors [17–19]. A common feature of the distance-based or information-based approach is that computational speed is very fast and requires less computational resources, but it can lead to high false positives.

To reduce the predictions of false positives, the phylogenetic profile combined with the species tree to explore the protein function, many methods have been proposed. For example, Pagel et al. proposed the relationship between the co-evolution of proteins based on the likelihood estimation [20, 21]. This method specifically estimates the direct coupling of two proteins to indicate that there is a direct co-evolution relationship between the two proteins. However, this method has obvious disadvantages as compared with the simple vector correlation as the measurement method, which requirements on computing resources and not suitable for the genomic level. Li et al. proposed a clustering of evolutionary conservation modules based on the hidden Markov model combined with phylogenetic and species evolutionary trees [7]. In this model, the author proposed an algorithm called CLIME that clusters known biological pathway proteins by the Dirichlet distribution and then infers the unknown proteins based on the evolutionary relationship of the species tree. The algorithm can accurately estimate the likelihood of predicted proteins in each evolutionarily conservative module. However, to estimate the zero hypothesis Gibbs sampling and simulating the annealing algorithm process must be run, which is much more time-consuming than the traditional co-evolutionary relationship measurement algorithm and take up a lot of computing resources. Dey et al. used phylogenetic profiles combined with a 177 eukaryotic tree constructed a homologous phylogenetic profile of humans to predict the function of human unknown genes [10]. Although this method extended more species tree to explored gene functions, the definition of protein interaction scores by phylogenetic tree deletion and transfer is not considered that the tree is rotatable. The different species ordering may bring completely different results that will lead to high false positives in predictions. The other methods proposed to reduce the rate of false positive predictions by normalization, such as singular value decomposition (SVD) [22, 23] and normalized phylogenetic profile (NPP) [12, 24, 25].

**Fig. 1:**
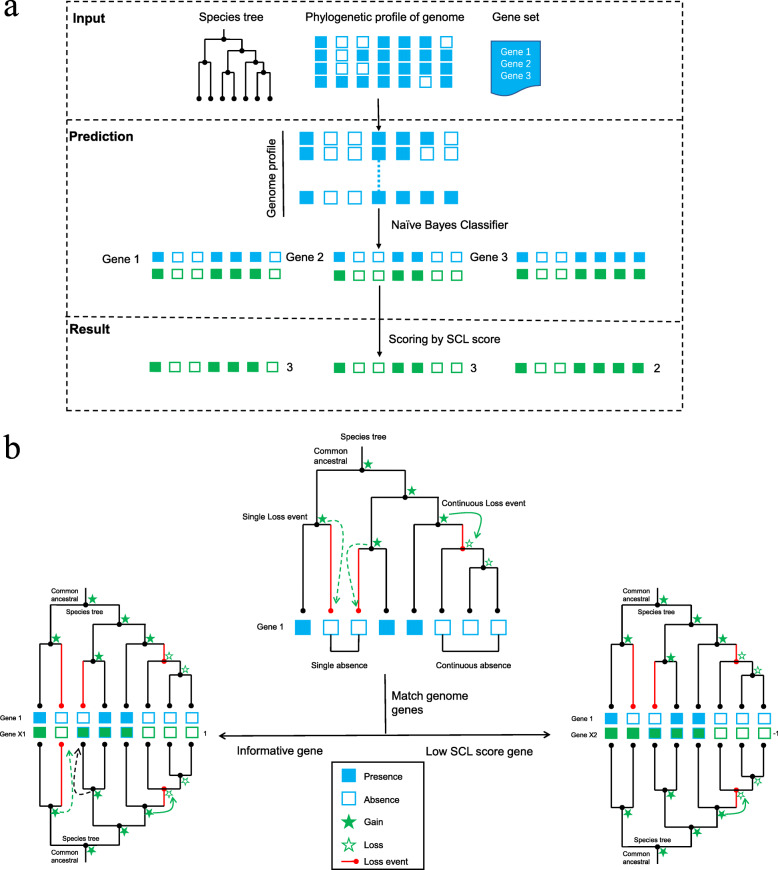
The schematic and algorithm of GFICLEE. **a** This program began with three inputs (1) species tree (2) binary phylogenetic profile (3) gene set. Then scan the genome profile for each input gene in the gene set with the Bayesian classification method. The final predicted the preferential genes with the same evolutionary pattern of the input gene set and scored by SCL score. **b** Mapping the presence and absence patterns of phylogenetic profile to gain and loss states in the species tree. The single absence map to species tree occurs single loss events (green dotted arrow) and continuous absence map to species tree occurs continuous loss events (green solid arrow). A penalty of 1 to genes in the genome which has the same single and continuous loss events with gene 1 (green solid and dotted arrow) and a penalty of − 1 to that have different single and continuous loss events

In this work, we propose Gene Function Inferred by Common Loss Evolutionary Events (GFICLEE) as a new method that infers genome-wide gene function. This work aims to discover new genes that are potentially involved in know biological pathways or cellular complexes. GFICLEE by mapping current gene profile presence and absence states to species tree with single and continuous loss events and finding which genes had the same loss events pattern by scanning the genome phylogenetic profile (Fig. 1). Notably, our algorithm is efficiently applied to genome-wide data, it’s about 30 times faster than the current best algorithm CLIME under the same-size gene sets. Aiming to address the challenge of the traditional algorithm always produce excessive false positive in the predictions. We designed GFICLEE with the tree-based method and the single and continuous loss score (SCL score) as scoring metrics that explore the genomic phylogenetic profile and infer which genes are informative shared the same evolutionary pattern with the input genes across genomic data (method). Strikingly, our algorithm is accurate, the cross-validations of Kyoto Encyclopedia of Genes and Genomes (KEGG) database [26], Gene Ontology (GO) database [27] and comprehensive resource of mammalian protein complexes (CORUM) database [28] database with constructed sensitivity and specificity curves manifest outperform current tree-based algorithm and distance/similarity method.

## Results

### GFICLEE: gene function inferred by common loss evolutionary events

We developed an algorithm named GFICLEE That base on binary phylogenetic profile and species tree (Fig. 1a). Users need to input three data (1) the gene set in the same biological pathway or biological complex, (2) a binary species tree, (3) the binary phylogenetic profile of all genes in this reference genome across the species. This species tree and binary phylogenetic profile can be defined by users. The input gene set belongs to the same biological pathway or cellular complex. When the user input gene set, the algorithm performs a genome-wide scan of each input gene searches the genes that have the same loss events as input genes. Firstly, GFICLEE classifies all genes in the genome profile into each input gene by the Bayesian classify method in the prediction section of Fig. 1a. Secondly, predicting the co-evolution proteins for each of the classified parts. Lastly, that predicted candidate gene scored by our defined SCL score (method). To reduce the amount of computation-intensive, our algorithm predicts informative genes began with the Naïve Bayesian method (Fig. 1b). Here we also compared the performance of other classification methods and the Naïve Bayesian method, such as Random Forest, Support Vector Machines, Nearest Neighbors. We used scikit-learn [29], a Python-based ML library to implement these algorithms (Additional file 1: Fig. S1). These performances show that the classification algorithm does not play a decisive role in the performance of the final prediction in our algorithm, so we choose the simple Naïve Bayesian method for classification.

GFICLEE has strongly assumed that genes are only gain once in the species tree but can be lost one or more times [30]. We use this assumption for the reconstruction of the ancestral state with the parsimony principle (Fig. 1b). In this way, we map the presence and absence patterns of the phylogenetic profile to gain and loss on the species tree topology. For GFICLEE algorithm, the “loss” flags in a binary profile of gene number correspond to changes of gene numbers from non-zero to zero in a species tree. Decreasing or unchanging gene numbers from non-zero to non-zero is classified into the “gain” category (Fig. 1b). The gene loss events in the species tree have two models, one is in the tree topology leaf node caused by genes that occur independent absence states in phylogenetic profile. We map the states to tree topology as an independent loss event and we defined this model as a single loss event (green dotted arrow). The other model is caused by genes that occur continuous absence states in phylogenetic profile. We map the states to tree topology as the continuous loss events and we defined this model as a continuous loss event (green solid arrow).

In our algorithm, a basic challenge was to define a scoring metric that effective to measure the genes in the genome that have the same evolutionary history in tree topology or presence and absence in profile patterns with input pathway genes. Here, we defined a phylogenetic co-evolution score (SCL score) (method) to distinguish the confidence of genomic genes. The SCL score is based on the genes that occur single and continuous loss events in tree topology. For example, Fig. 1b match genome genes by this method, if the gene x in the genome has the same single or continuous loss event with input genes, gene x is penalized 1 point each time (green solid and dotted arrow). Conversely, there is no match to a consistent loss event that penalty is − 1 (black dotted arrow). By this method, we measure all genes in the genome phylogenetic profile by the same scale, the high SCL scores represent informative genes in the predicted result.

### The pathway genes occur gain event on the same common ancestor

**Fig. 2:**
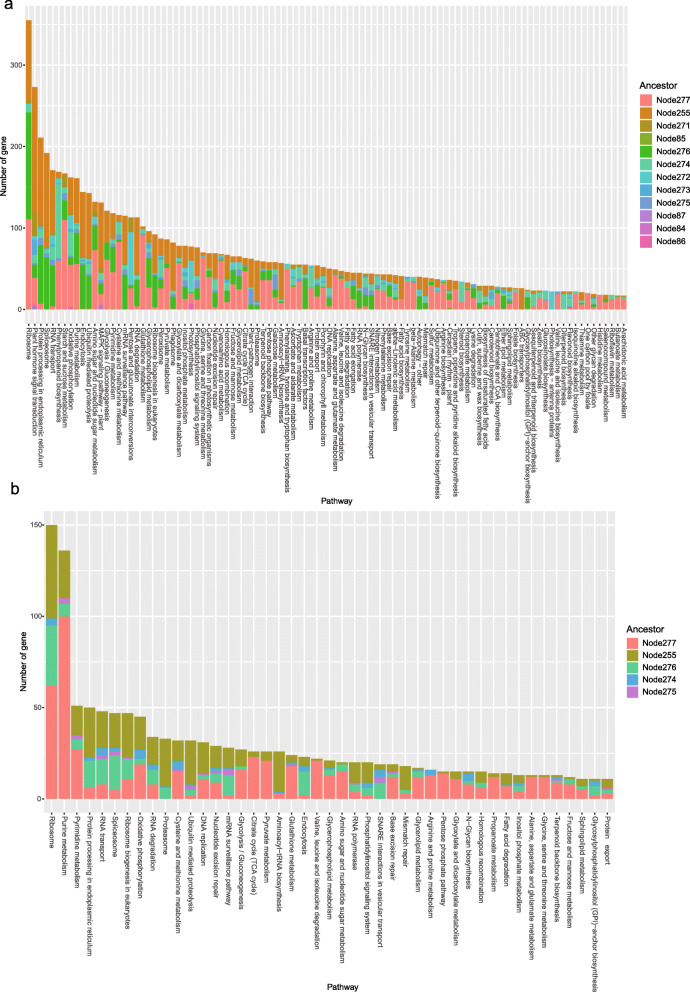
The common ancestor of genes in different pathways. **a** The distribution of gene’s common ancestor in *A. thaliana* pathways. **b** The distribution of gene’s common ancestor in *T. brucei* pathways

Our algorithm scans the input genes one by one in the genome-wide phylogenetic search for genes that have the same evolutionary pattern. Instead of clustering the input genes and then predicting the informative genes, the goals just like the CLIME algorithm [7]. Our methodological approach is based on gene phylogenies to infer genes that have the same evolutionary in all genomes. GFICLEE scan all genes in the genome and filter gene by the same common ancestor since we scan all genes in the *Arabidopsis thaliana (A. thaliana)* and *Trypanosoma brucei* (*T. brucei*) dataset respectively and record genes occur gain node event (Fig. 2a, b). The distributions of gain nodes are showing that genes in one pathway can be clustered into different nodes. In the *A. thaliana* genome genes, all pathway genes in the figure are clustered into 12 different nodes, which indicates that the genes have the same evolutionary pattern with the common ancestor in the same node (Fig. 2a). This phenomenon is more obvious in *T. brucei* that has a smaller genome, and these genes are clustered in 5 different nodes only (Fig. 2b). We also test the pathway in CORUM database (Additional file 1: Fig. S2a), GO database (Additional file 1: Fig. S2b) and KEGG database (Additional file 1: Fig. S2c) also shows that most genes gain event occur in the common ancestor. The distributions of the common ancestor showing that in each pathway only 10% of the genes have the same common ancestor and cover 86.1% in *A. thaliana* 75.6% in *T. brucei*. It also indicates that it is necessary to perform a genome-wide scanning of every input gene. Although genes in one pathway always occur gain event in a common ancestor, their genes in one pathway usually cluster in a different node. For example, the genes in the pathway “Arachidonic acid metabolism” clustered into 4 different nodes and genes in the “Ribosome” pathway clustered into 6 different nodes (Fig. 2a). it indicates that genes in one pathway first occur gain event can form different nodes that lead to genes functions appeared later or earlier than input genes. There has a strong assuming that the genes with similarity function appear in the common ancestor for GFICLEE (Additional file 1: Fig. S3a (1)). Hence, we set GFICLEE to predict genes that have a similar function from the common ancestor as the default parameter. For gene functions that appear later or earlier than the input gene (Additional file 1: Fig. S3a (2,3,4)), GFICLEE also provided an interface for users to choose from. We test the performance of different cases (Additional file 1: Fig. S3b) show that The performance of search node form input genes node is best (Additional file 1: Fig. S3b (1)) than the other three settings. Interestingly, setting the search node earlier than the input genes node (Additional file 1: Fig. S3b (4)) is very similar to the search node from the input genes node (Additional file 1: Fig. S3b(1)).

### Predictive performance of GFICLEE

**Fig. 3:**
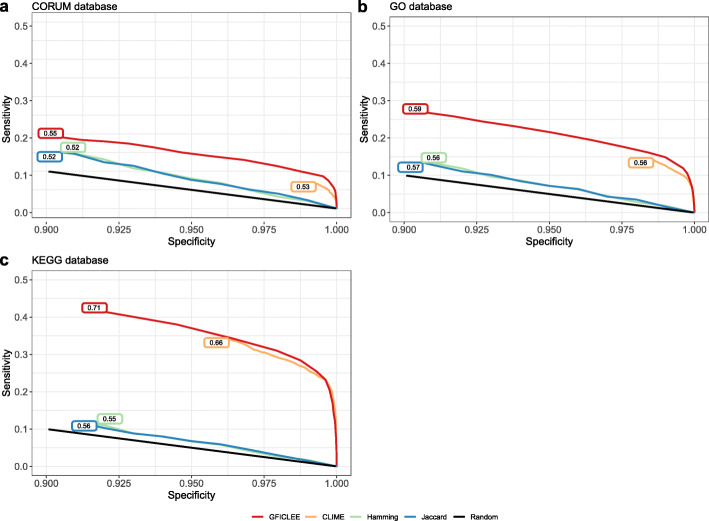
The performance of GFICLEE compares with existing approaches by three databases. The sensitivity and specificity curves constructed by 5-fold-leave-half-out cross-validation are shown in **a** CORUM database, **b** GO database and **c** KEGG database with the human genome

We compared our algorithm with a tree-based algorithm CLIME (version 1.1) [31], the similarity-based method Jaccard and distance-based Hamming distance by using 5-fold-leave-half-out cross-validation on the biological pathway or cellular complex. To validated the robustness of our algorithm, we used the human genome with the biological pathways and cellular complexes from CORUM (1056 complexes) [28], KEGG (116 pathways) [26] and GO (911 gene sets) [27] databases. For each pathway, complex and gene set we constructed sensitivity and specificity curves to show that all methods are substantially better than random chance at all specificity values (Fig. 3). In comparison to CLIME, GFICLEE had enhanced sensitivity upon decreasing the specificity (1-specificity was increasing) in the CORUM and GO database. For example, the specificity from 0.993 to 1.00 in CORUM complex (Fig. 3a) and from 0.986 to 1.00 in GO gene set (Fig. 3b) GFICLEE have higher sensitivity, showing a noticeable improvement. Interestingly, we found that GFICLEE performed exceptionally better than the other three methods in the GO and CORUM databases, while the KEGG database showed similar performance with CLIME (Fig. 3c). This indicates that GFILCEE is more robust as the test dataset increases. Specifically, the CLIME algorithm using log-likelihood Rate (LLR) scoring predicted genes with threshold greater than 0 (Test ROC curve with thread from 0 to 60) and our algorithm does not have artificially set thresholds (Test ROC curve with thread from − 19 to 34). Our algorithm can cover more genes in the entire genome and had a wider threshold. In the verifications of the three databases, our algorithm is still better than the CLIME algorithm at threshold 0 of the CLIME algorithm. For example, As of the LLR scoring threshold of 0, the sensitivity of CLIME was 0.068, 0.141 and 0.358, whereas the sensitivity of GFICLEE was 0.096, 0.161 and 0.360 in CORUM, GO and KEGG dataset respectively showing the GFICLEE had higher sensitivity than CLIME in threshold lower bound 0. We calculated the area under the curve (AUC), we observed that the AUCs of GFICLEE were large (0.550, 0.594, 0.713) in comparison to CLIME and hamming/Jaccard method in three database gene set (Fig. 3a, b, c). The test results show that tree-based algorithms do perform better than traditional distance or similarity methods in predicting gene function.

The cross-validation shows that our algorithm is robust and gives good prediction results across genome-wide. We also validated our algorithm with the different genomes, we papered the *A. thaliana* phylogenetic profile and the input dataset to download from the KEGG database (Release 88.1) [26]. The sensitivity and specificity curve showed that our algorithm is still practical in the model organism *A. thaliana* (Additional file 1: Fig. S4a). Moreover, to further verify the universal applicability of our algorithm in eukaryotic genomes, we used *Trypanosoma brucei (T. brucei)* genomes that belonged to protists to validate our algorithm (Additional file 1: Fig. S4b). The validations of the three pathway datasets from different databases, *A. thaliana* and *T. brucei* genome demonstrated that the robustness and practicability of our algorithm.

### Algorithm implementation and computational efficiency

**Fig. 4:**
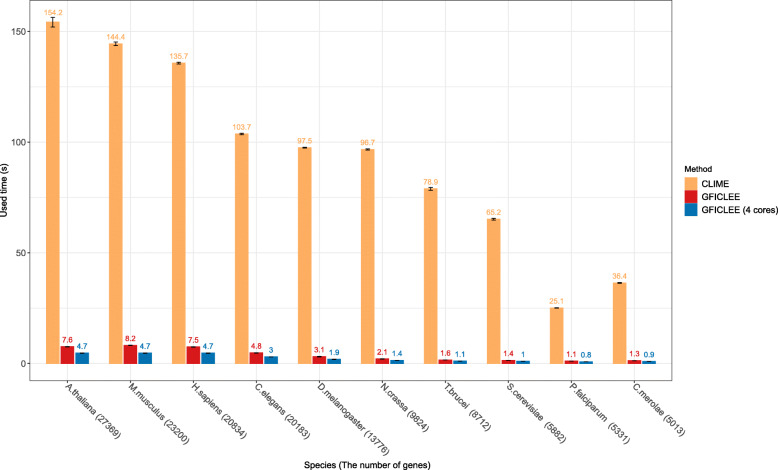
The computational time of GFICLEE compares with other methods with different genomes. The computational time of our algorithm compares with CLIME algorithm with 10 different scale genomes. The yellow histogram represents CLIME, the red histogram represents our algorithm GFICLEE and the blue histogram represents GFICLEE that implemented parallel computing method (4 cores)

We implemented GFICLEE in Java programming language and Supported Linux, Windows, and Mac OS platforms (https://github.com/yangfangs/GFICLEE1.0). We compared the computational time between our algorithm with CLIME algorithm by using different scale genome-wide datasets (Additional file 2: Table S2). About that genomes, 5 of them are more than 10,000 genes, and the other five not more than 10,000 genes. We recorded the computational time with the same input gene set contains 17 genes. The computational time of our algorithm showed that our algorithm runs very fast for small genomes. For example, our algorithm scans *T. brucei* (8712 genes), *S. cerevisiae* (5882 genes), *Plasmodium falciparum* (*P. falciparum*) (5331 genes) and *Caenorhabditis elegans* (*C. merolae*) (5013 genes) only used 1 s. However, the CLIME algorithm takes 90, 68, 25 and 37 s to complete the prediction, respectively (Fig. 4). As the scale of genomic data increases, our algorithm is still faster than the CLIME algorithm in terms of computational speed. Such as, in *A. thaliana* genome (27,369 genes) the computational time about 30 times faster than CLIME algorithm, test in *Mus musculus* (*M. musculus*) (23,200 genes), *Homo sapiens* (*H. sapiens*) (20,834 genes), *C. elegans* (20,183 genes) and *Drosophila melanogaster* (*D. melanogaster*) (13,776 genes) genomes the computational time average of 24 times faster than CLIME algorithm (Fig. 4). Additionally, we designed GFICLEE with parallel computation, using 4 cores to scan the ten genomes in parallel that was very significant for the improvement of computational efficiency (Fig. 4). We test 5-fold-leave-half-out cross-validation for three different datasets (KEGG, GO, CORUM) and recorded the running time that shows in Additional file 1: Fig. S5 and Additional file 2: Table S1. For three datasets, CLIME used 1856, 1389, 337 min and our algorithm only takes 128, 117 and 19 min running each test dataset, respectively. These results suggest that our algorithm has very high computational efficiency and can retrieve highly informative genes from the genome in a very short time.

### Validated algorithm by cilia components and WASH complex

**Fig. 5:**
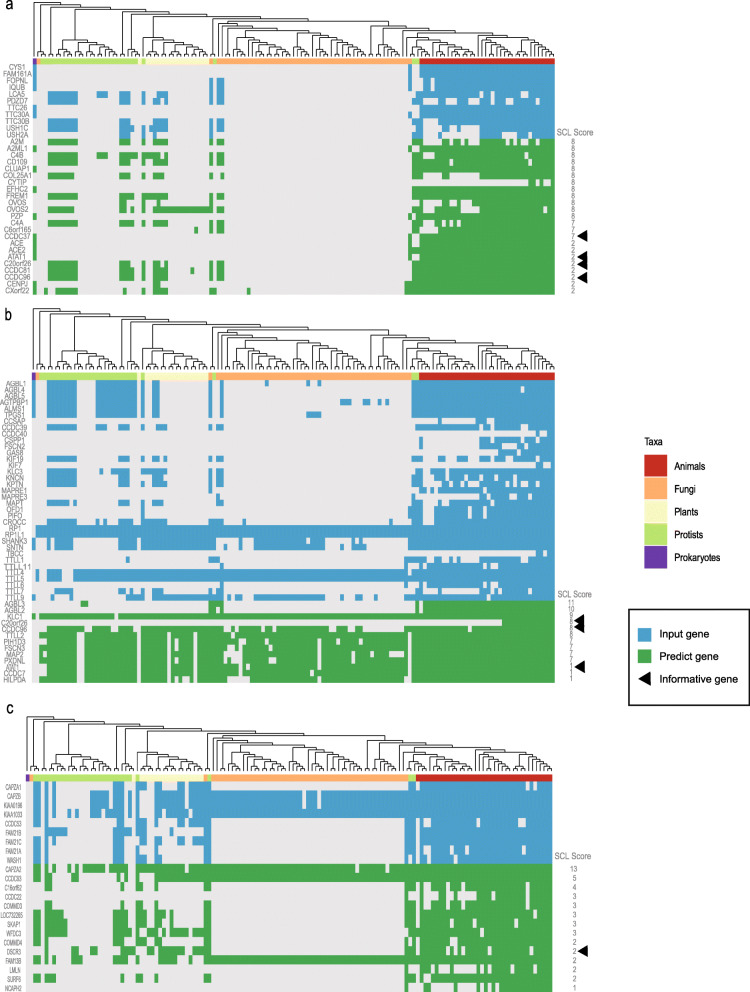
The prediction of cilia components and WASH complex. **a** The predictions of cilia module in cilia components. **b** The predictions of cytoskeleton module in cilia components. **c** The predictions of WASH complex

To validate the ability of our algorithm to predicting new gene members associate with the known biology pathway and complex. We choose cilia components and WASH complex as a study case, We applied GFICLEE to the module that contains 11 genes (Fig. 5a) named cilia that was an important organelle of human that well-studied and successful in previous phylogenetic profiling studies [7, 10, 11, 32]. The input 11 genes in the cilia module had a relatively consistent absence pattern in the phylogenetic profiles, such as the occurrence of large-scale absence events in fungi, which is consistent with the description of the review by Carvalho-Santos et al. [33] (Fig. 5a). We show the predicted genes at the top of scored by SCL score. Here the gene named CCDC37 was of particular interest to us because not only it was had highly SCL score of our predictions but also experiments have confirmed that this gene was a component of the cilia module [10]. There are also have informative genes of the predictions, such as, the genes named ATAT1, C20orf26, CCDC96 with the SCL score more than zero, which also belong to cilia components was proved by experiments [10]. Next, we applied GFICLEE to the cytoskeleton module of cilia, which contains 35 genes (Fig. 5b). The GFICLEE also predicted a high SCL score of genes, such as C20orf26, CCDC96 and ATAT1, CCDC104 with SCL score more than zero are completely uncharacterized. Moreover, the WASH complex has accumulated a wealth of research results, such as involved in endosome trafficking [34] and regulating endosome morphology [35]. Here we input the WASH complex gene set contains 9 genes into GFICLEE (Fig. 5c). In the prediction of genes that include DSCR3 with 2 points that recent experiments revealed have strongly physically associated with the WASH complex [10]. Other predicted genes, such as CAPZA2 had top SCL scores (SCL score = 13) that are completely uncharacterized (Fig. 5c). These predictions demonstrate the ability of our algorithm to predict biological pathways or complexes at the genome-wide level.

### The horizontal genes transfer (HGT) effect on GFICLEE

We inferred the genes common ancestral by naïve Dollo parsimony, which are very sensitive to horizontal transfer genes. When inferring common ancestors, HGT events often have a large impact on inferred results [36, 37]. The HGT events might lead to the false presence events in the phylogenetic profile, which can be caused by falsely inferred common ancestral and consequently higher loss events estimates. To verify the HGT events that impact the robustness of our algorithm, we revised the phylogenetic profile by inferred the suspected HGT events (method). By phylogenetic profile approach, we detected 3732 genes might arrive by HGT events in human phylogenetic profile, in the total human genome genes (20834) the HGT events occur in Animals group (3.9%) less than the other three subgroups (Fungi %4.8, Plants 4.5%, Protists 4.7%) (Additional file 1: Fig. S6 a). This indicated that HGT events occur more rarely in human animals. After that, we test GFICLEE by revised phylogenetic profile and compared it with the origin result in three different databases. The sensitivity and specificity curve performance show that the test results of the revised phylogenetic profile are consistent with the original results (Additional file 1: Fig. S7). The example genes of HGT events detected by the revised filter method are shown in Additional file 1: Fig. S6 b. The results indicate that our algorithm uses naïve Dollo parsimony to infer the ancestor state, which has little effect on the occurrence of HGT events and illustrates the robustness of our algorithm. Here we also test GFICLEE by removing suspected genes of HGT from the phylogenetic profile and test data set and our algorithm also has the best performance (Additional file 1: Fig. S8). The performance of GFICLEE suggests that our approach centered around the use of naïve Dollo parsimony to infer gene function for all genes in all genomes, is robust to the effect of HGT, variable genome size and genome completeness, factors that strongly affect gene function inferred.

## Discussion

The GFICLEE algorithm based on the parsimony principle. By this principle, mapping the gene’s presence and absence states to gain and loss states in the species tree topology. We use both the phylogenetic profile presence/absence state pattern information (Bayesian classification) and the gain/loss and information of the species tree (single and continuous loss events) to infer gene function. This avoids the Hamming/Jaccard method that merely considers the phylogenetic profile presence/ absence patterns leads to excessive false positive results. Due to our algorithm inferred ancestral states by parsimony principle, it is observed that our algorithm was only applied to eukaryotes and it performed was not well for prokaryotes. Because using parsimony methods to accurately inferred ancestral states requires the rates of changes are low [38]. This requirement is met in eukaryotes because the HGT events between different non-endosymbiotic origin are rare but in prokaryotes are very common [39]. Although our algorithm is still very robust performance with HGT events, we still recommend GFICLEE for eukaryotic genome prediction.

To reduce false positives in gene function predicted, we scored the predicted genes with our defined SCL score. However, we know that our defined SCL score can generate false-negative results in predictions, which genes have co-evolution relativity but predicted the result with lower scores. In the review [40], the author illustrated four scenarios for the evolution of states of characters gene X and Y. We describe the four evolutionary relationships corresponding to our algorithm as shown in Additional file 1: Fig. S9. We argue that two scenarios (Additional file 1: Fig. S9a, b) provide good evidence for the functional relationship among gene X and Y because it corresponding to our algorithm model single/continuous loss events and continuous loss events only. Our defined SCL score to measure the predicted genes can give the scenarios of “replicated co-distribution” and “Darwin’s scenario” with a high SCL score. For the scenario of “Replicated bursts” and “Unreplicated burst” (Additional file 1: Fig. S9 c, d), the GFICLEE can give a lower SCL score than the previous two scenarios. In our prediction result, if there have the latter two scenarios, it will be predicted as a false negative result. The SCL score also has problems that it does not solve all four scenarios that are described in the review.

We designed GFICLEE was a tree-based method algorithm, input a correct species tree is very necessary for our algorithm. Because we used the Dollo parsimony approach for the ancestral state reconstruction, the topology of the species tree has a great influence on the determination of the common ancestor state that occurs single/continuous loss event. Here, we test our new method with four different reconstructed tree software (FastTree [41], IQ-Tree [42], MEGA [43] and PhyML [44]) and random topology tree (generate by the R package “ape” [45]) (Additional file 1: Fig. S10). The results show that our new method is robust to various software reconstruct tree, but the random topology tree gets the worst performance. It suggests that a correct species tree is very necessary for our new method. Our algorithm used a homology-based binary phylogenetic profile (derived from BLASTP with expect threshold), multiple reasons cause the errors in binary phylogenetic inference. For example, When genome sequencing, there had many technical difficulties in it [46], using faulty protein predictions [47], there had some bias when we detecting sequence similarity [48]. It is also crucial to input an accurate phylogenetic influence on the predicted results. In this study, we use BLASTP (threshold with E-value < 10–3) to generate homology-based binary phylogenetic profile to test our new algorithm. We also compared our new method with different threshold build binary phylogenetic profile (Additional file 1: Fig. S11). The result suggests that the appropriate threshold to detect orthologs and build phylogenetic profile is necessary to our new algorithm. Our algorithm aims to provide a fast and efficient algorithm for searching genes with similar functions across the genome. So, we did not optimize the phylogenetic profile like singular value decomposition (SVD) [22, 23]. Therefore, when the users used GFICELEE, they need to input the correct phylogenetic profile and species tree to get better prediction results.

The different taxon sampling may be impacts the prediction performance of GFICLEE. we test the GFCILEE method by different subtree that the random sampling are collected from 138 species tree [49]. We extract species from each phylum classification at a ratio of 20, 40, 60, and 80% to generate subtrees by ETE3 software [50]. For example, the ratio of 20% contains 15 animals, 6 plants, 22 fungi and 12 protists. Moreover, we also test the performance of GFICLEE with different subtrees generate by phylum (29 protists, 56 fungi, 38 animals and 16 plants). We test the performance of GFICLEE by different scale taxon sampling subtree with KEGG database (Additional file 1: Fig. S12). In the result, we can see that the 100% species taxon has the best performance and the worst performance is 20% subtree. As the number of sampling species increases, the prediction performance gets better and better. However, the performance of the phylum-based subtree is poor. The best is the subtree contains 29 protists that performance results are similar to a subtree (20%), suggesting that the dense taxon sampling can compensate for the influence of misidentification of the species tree.

The GFICLEE is a very fast algorithm mapping the phylogenetic profile presence and absence states to gain and loss states in a species tree topology. GFICLEE using an algorithm based on the parsimony principle and complexity of *O*(*n*) for each gene, where *n* is the number of nodes in the tree, however CLIME algorithm of the complexity of *O*(*Sn*^2^) per MCMC iteration, where *S* is the number of species, and *n* is the number of genes in the input gene set [7]. CLIME calculation time increases with the square of the input gene, while our algorithm calculates the time for each gene is constant. Compared to CLIME, our algorithm does not require MCMC iteration and Gibbs sampling. These two processes are very time-consuming and the Gibbs sampling process does not allow parallel calculations. Our algorithm does not need to worry about this problem at all, which gives our algorithm a huge advantage in computing speed.

## Conclusions

Here we introduced a tree-based method, GFICLEE, for explored genomic data predicted new gene potential involved in known biological pathways or cellular complexes. The phylogenetic profile is not only widely used for protein interaction exploration, but also very active in other fields such as, annotate genomes [51–54], protein subcellular localization [55], chromosomal location analysis [56], Identification of small RNA [12], found human disease locus [25] In this study, GFICLEE can input any binary phylogenetic relationship and ultrafast scanning whole genomic genes reveal genes relationship, which also makes it possible for GFICLEE to complete the genome-wide annotate gene functions of new sequencing organisms in a very short time. We expect that future versions of GFCILEE may accept more different binary phylogenetic matrix, and provide more favorable tools for exploring the function of genes at the omics level.

## Methods

### GFICLEE algorithm

#### Step 0: mapping

For each gene, GFICLEE assumes that genes are only gain once in the species tree but can be lost one or more times [30], based on this methodological approach reconstructed ancestral state with the parsimony principle. GFICLEE using a deep first search graph algorithm build the lowest common ancestral (gain node) [57]. After the lowest common ancestral was determined, GFICLEE mapping each phylogenetic profile presence and absence states to species tree and recorded by single and continuous loss events. In this way, GFICLEE recorded the loss events and the lowest common ancestral in evolutionary history for every gene in the genome.

#### Step 1: classify

For each input gene, GFICLEE uses the Naïve Bayes Classifier [58] to classify all genes in the phylogenetic profile by each input genes gain/loss pattern, so that genes with similar patterns can be classified to the corresponding predicted cluster. This can avoid the computational time-consuming problems caused by scanning of all genes in the phylogenetic profile for each input gene. For all predicted genes that have been classified, GFCLEE infers candidate genes with similar evolutionary history based on the same lowest common ancestor information (default).

#### Step 2: prediction

GFICLEE prediction informative genes form candidate genes by single or continuous loss event. The candidate genes have the same single or continuous events with input genes that infer to be informative genes. GFICLEE scoring all candidate genes in each classification by SCL score. The high SCL score genes are informative shared the same evolutionary pattern with the input genes across the genome.

### Definition of SCL score

For all predicted genes in the genome, we scored by single continuous loss score (SCL score) that we defined as a measure score. The SCL score of gene A and gene B we defined as follow:

$$ \mathrm{SCLscore}\left(\mathrm{A},\mathrm{B}\right)=\sum \limits_{\begin{array}{c}i\in loss\ branch\ of\ A,\\ {}i\in loss\ branch\ of\ B\end{array}}\left\{ anc\left({a}_i\right)- desc\left({a}_i\right)\right\}-\sum \limits_{\begin{array}{c}i\in loss\ branch\ of\ A,\\ {}i\notin loss\ branch\ of\ B\end{array}}\left\{ anc\left({a}_i\right)- desc\left({a}_i\right)\right\}\ (1) $$

Here, the eq. (1), *a* denotes arbitrary gene, *anc*(*a*_*i*_), *desc*(*a*_*i*_) ∈ {0, 1} denotes the ancestral state and descendant state for the branch *i* inferred for the gene A and B profile respectively. The loss branch includes a single loss branch and a continuous loss branch. The $$ {\sum}_{\begin{array}{c}i\in loss\ branch\ of\ A,\\ {}i\in loss\ branch\ of\ B\end{array}}\left\{ anc\left({a}_i\right)- desc\left({a}_i\right)\right\} $$ denotes loss score calculate by gene A and gene B that have the same loss branch which penalty of 1. The $$ {\sum}_{\begin{array}{c}i\in loss\ branch\ of\ A,\\ {}i\notin loss\ branch\ of\ B\end{array}}\left\{ anc\left({a}_i\right)- desc\left({a}_i\right)\right\} $$ denotes loss score calculate by gene A but gene B did not have the same loss branch.

### Naïve Bayes classifier (NBC)

To predict the genes in phylogenetic profile are belong to which input genes, we trained a Naïve Bayes Classifier. The Naïve Bayes Classifier (NBC) is a machine learning probabilistic classifier based on learns from training data and then predicting the class of the given instance with the highest posterior probability [58]. For the input genes g, let g ∈ {*C*_1_, …, C_*g*_} denotes a set of g classes, if the input gene sets have different phylogenetic patterns. Here we predict a gene *X*_*t*_ from genome phylogenetic profile that is an unknown class to G. Based on the Bayes’ theorem (also called the Bayes rule), According to it the class ci for *X*_*t*_ should is the one, which maximizes the probability:

$$ \mathrm{P}\left({C}_i|{X}_t\right)=\frac{P\left({C}_i\right)P\left({X}_t|{C}_i\right)}{P\left({X}_t\right)}\ (2) $$

Where *C*_*i*_ is the ith class we total input gene g, *X*_*t*_ is a binary vector in phylogenetic profile. For each gene profile across N species equal it has N feature because of f_*i*_ are independent given the class then *P*(*X*_*t*_| *C*_*i*_) can be decomposed into the product *P*(*x*_1_| *C*_*i*_) ∗ , …, ∗ *P*(*x*_*N*_| *C*_*i*_). Thus, the predicted class *C*_*i*_ belong to the one, which maximizes the probability:

$$ \mathrm{P}\left({C}_i|{X}_t\right)=\frac{P\left({C}_i\right)}{P\left({X}_t\right)}\ \prod \limits_{j=1}^NP\left({x}_j|{C}_i\right)\ (3) $$

Estimating the probability *P*(*X*_*t*_) is unnecessary because it is the same for all classes *C*_*i*_, we only maximize the probability:

$$ \mathrm{P}\left({C}_i|{X}_t\right)\propto P\left({C}_i\right)\ \prod \limits_{j=1}^NP\left({x}_j|{C}_i\right)\ (4) $$

### Pathways and genome data

The KEGG biological pathways, GO cellular component dataset, human genome phylogenetic profile and the 10 genomes for test computational time with 138 species tree and corresponding phylogenetic profiles are the same with CLIME paper description, downloaded from http://gene-clime.org/ [7]. The CORUM human biological pathways and complexes were downloaded from CORUM database (02.07.2017 Release) [28]. The *A. thaliana* metabolic and signaling pathways were downloaded from the KEGG pathway database (Release 88.1) with three large terms “metabolism”, “cellular processes” and “Genetic information processing” [26]. BLASTP [59] was used to comparing 27,396 *A. thaliana* protein sequences with the selected species. We set the BLASTP E-value 0.001 as the threshold (E-value < 0.001) constructed the homology matrix, in which 1 denoted those homologies of *Arabidopsis thaliana* proteins found in the corresponding organism, otherwise 0. The *T. brucei* metabolic and signaling pathways were downloaded from KEGG pathway database (Release 88.1) and remove the terms of “Human Diseases”,” Brite Hierarchies”,” Not Included in Pathway or Brite” [26].

### 5-fold-leave-half-out cross-validation and construct ROC

The human biological pathways and cellular complexes contain 1056 protein complexes from the CORUM database [28], 116 biological pathways from KEGG database [26] and 911 cellular component database from GO cellular component dataset [27]. We compared our algorithm to CLIME software using 5-fold-leave-half-out cross-validation. For each pathway, we separated it into two parts, one as the input genes, and the other as validated genes and executed this separated 5 times. About CLIME software we carry out with the description in that paper, for a range of LLR thresholds, sensitivity was calculated as the percent of the genes correctly recovered in any ECM+ derived from the leave half out input gene set and specificity was calculated as the percent of non-pathway genes correctly absent from all ECM+ derived from the leave half out input gene set [7]. About our algorithm, we used the same method to calculate sensitivity and specificity for a range of SCL score thresholds. For each threshold, we can get the number of TP true positives (TP) and true negative (TN) represented the positive and negative predicted genes above this threshold. In contrast, the false positive (FP) and false-negative (FN) were detected as positive and negative predicted genes below this threshold. The sensitivity and specificity were calculated by the following equations.

$$ \mathrm{Sensitivity}=\frac{TP}{TP+ FN}\ (5) $$

$$ \mathrm{Specificity}=\frac{TN}{TN+ FP}\ (6) $$

### Distance and similarity method

We carry out the distance and similarity method to predict the informative genes and validated by a 5-fold-leave-half-out cross-validation method in three human biological pathways and cellular complexes. For this method, we calculated the two binary profile distances between each input gene with all genes in the genome phylogenetic profile and then we choose the minimum distance or similarity as the predicted genes. We carry out the Hamming and Jaccard method by SciPy [60] that a Python-based ecosystem of open-source software.

### Running environment

Here we compare our algorithm GFICLEE with CLIME software (version 1.1) in computation time with different datasets. Firstly, we used the human genome (20,063 genes) with three different databases are CORUM, GO and KEGG. We control the comparisons in the same scale of genomes and pathway genes set with different databases (Additional file 2: Table S1). Tested this dataset used 5-fold-leave-half-out cross-validation method within 10 cores (Intel Xeon E5620 2.4GHz, 48Gb memory) parallel processing running environment. Secondly, we tested computation time in different organisms (10 genomic data) with the same input gene set. (Additional file 2: Table S2). This test with the same input gene set contains 17 genes in different genomes run the software by a personal computer (i7–4790 3.6GHz, 16GB memory) with Fedora Operating System.

### Filter the HGT of individual genes

We infer suspected genes of HGT events by the phylogenetic profile approach. This method’s basic theory is an isolated presence gene that occurred in the absence of homology in the closely related species, that event is indicated that the isolated presence gene might have arrived via HGT event [61]. To find which genes have an HGT event we divided the 138 species into five supergroups by level of phylum: Animals (37), Plants (16), Fungi (56), Protists (29). Then we scan each gene in the phylogenetic profile that if isolated presence genes occurred in any of five subgroups we revised those genes as absence stated. The performance of GFICLEE showed by sensitivity and specificity curves display. At the same time, we also tested our method by removing the suspected genes of HGT from the phylogenetic profile and the performance of GIFLEE showed by sensitivity and specificity curves. In addition, the filter HGT method are Integrate to GFILCEE and the users can explore gene function by “-rm” and “-rv” option.

## Supplementary Information

**Additional file 1: Supplementary Fig. S1.** The performance of different classifies methods. The performance of naïve Bayesian method compares with Random Forest, Support Vector Machines and Nearest Neighbors classify methods. In the figure, NB: naïve Bayesian, SVC: Support Vector Machines classification, NC: Nearest Neighbors classification and RF: Random Forest classification. **Supplementary Fig. S2.** The common ancestor of genes in different databases. a) The common ancestor of genes in the human pathway with CORUM database. b) The common ancestor of genes in the human pathway with GO database. c) The common ancestor of genes in the human pathway with KEGG database. **Supplementary Fig. S3.** The comparison of genes functions appeared in later or earlier than input genes. a) Setting the search node is same with input genes node (1), Setting the search node later and earlier than input gene node (2), Setting the search node later than input genes node (3), Setting the search node earlier than input genes node (4). b) The performance of setting the different search gain node. **Supplementary Fig. S4.** The performance of GFICLEE compares with existing approaches by different genomes. a) The *A. thaliana* metabolic and signaling pathways. b) The *T. brucei* metabolic and signaling pathways. **Supplementary Fig. S5.** The computational time of three different databases. The test used the human genome with the GO database that contains 911 pathways, CORUM contains 1056 pathways and KEGG with 116 pathways. The test parallel running with 10 cores for CLIME and GFILEE software, respectively. **Supplementary Fig. S6.** The HGT events occurred in the human phylogenetic profile. a) The HGT events occurred in each subgroup. b) The example of the revised HGT profile. **Supplementary Fig. S7.** The performance of GFICLEE by phylogenetic profile compares with the revised phylogenetic profile in three databases. **Supplementary Fig. S8.** The performance of GFICLEE compares with CLIME by the phylogenetic profile that removes suspected HGT genes. **Supplementary Fig. S9.** GFICLEE algorithm corresponding four scenarios for the evolution of states of characters gene X and Y. a) The replicated co-distributions contain single and continuous loss events. b) Darwin’s scenario only contains continuous loss events. c) and d) The scenarios of Replicated bursts and Unreplicated burst, respectively. **Supplementary Fig. S10.** The performance of GFICLEE with different software reconstruct species tree and random topology tree. FastTree, IQ-TREE, MEGA_ML and PhyML are maximum likelihood method reconstruct species tree. The MEGA_ML is the Neighbor-Joining method reconstruct species tree. **Supplementary Fig. S11.** The performance of GFICLEE with different threshold generate phylogenetic matrix. The various BLASTP threshold to (E-value < 10–2, E-value < 10–3, E-value < 10–4 and E-value < 10–5) generate ortholog matrix and test the performance of GFICLEE. **Supplementary Fig. S12.** The taxon sampling effect on the performance of GFICLEE. We extract species from each phylum classification at a ratio of 20, 40, 60, and 80% to generate subtrees. The contains each phylum only also extracted for the performance test.

**Additional file 2: Supplementary Table S1.** The datasets of the human genome with KEGG database, CORUM database and GO Database. **Supplementary Table S2.** The datasets of 10 different scale genomes.

## Data Availability

GFICLEE is free available as Java command line software under the GPL-3.0 License license (https://github.com/yangfangs/GFICLEE1.0), the reproducible benchmarking codes available from https://github.com/yangfangs/test_GFICLEE. The data comes from KEGG, GO, CORUM databases, which are all public open platforms. KEGG (https://www.genome.jp/kegg/); GO (http://geneontology.org/); CORUM (https://mips.helmholtz-muenchen.de/corum/#). Details on the 10 genomes and 138 species tree and corresponding phylogenetic profiles downloaded from http://gene-clime.org/.

## Abbreviations

*S. cerevisiae*: *Saccharomyces cerevisiae*
*D. melanogaster*: *Drosophila melanogaster*
*C. elegans*: *Caenorhabditis elegans*
*A. thaliana*: *Arabidopsis thaliana*
*M. musculus*: *Mus musculus*
*H. sapiens*: *Homo sapiens*
*N. crassa*: *Neurospora crassa*
*T. brucei*: *Trypanosoma brucei*
P. falciparum: *Plasmodium falciparum*
C. merolae: *Cyanidioschyzon merolae*
KEGG: Kyoto Encyclopedia of Genes and Genomes
GO: Gene Ontology
CORUM: Comprehensive resource of mammalian protein complexes
AUC: Area under the curve
HGT: Horizontal genes transfer
SVD: Singular value decomposition
NBC: Naïve Bayes Classifier
SCL: Common single and continuous loss
NPP: Normalized phylogenetic profile
ROC: Receiver operating characteristic
MCMC: Markov chain Monte Carlo
RNA: Ribonucleic acid
