# Supplementary material for: GFICLEE: ultrafast tree-based phylogenetic profile method inferring gene function at the genomic-wide level
Source: BMC Genomics. 2021 Oct 29;22:774. doi: 10.1186/s12864-021-08070-7 (PMC8557005; doi:10.1186/s12864-021-08070-7)
Supplement: Supplementary file 1 — Additional file 1: Supplementary Fig. S1. The performance of different classifies methods. The performance of naïve Bayesian method compares with Random Forest, Support Vector Machines and Nearest Neighbors classify methods. In the figure, NB: naïve Bayesian, SVC: Support Vector Machines classification, NC: Nearest Neighbors classification and RF: Random Forest classification. Supplementary Fig. S2. The common ancestor of genes in different databases. a) The common ancestor of genes in the human pathway with CORUM database. b) The common ancestor of genes in the human pathway with GO database. c) The common ancestor of genes in the human pathway with KEGG database. Supplementary Fig. S3. The comparison of genes functions appeared in later or earlier than input genes. a) Setting the search node is same with input genes node (1), Setting the search node later and earlier than input gene node (2), Setting the search node later than input genes node (3), Setting the search node earlier than input genes node (4). b) The performance of setting the different search gain node. Supplementary Fig. S4. The performance of GFICLEE compares with existing approaches by different genomes. a) The A. thaliana metabolic and signaling pathways. b) The T. brucei metabolic and signaling pathways. Supplementary Fig. S5. The computational time of three different databases. The test used the human genome with the GO database that contains 911 pathways, CORUM contains 1056 pathways and KEGG with 116 pathways. The test parallel running with 10 cores for CLIME and GFILEE software, respectively. Supplementary Fig. S6. The HGT events occurred in the human phylogenetic profile. a) The HGT events occurred in each subgroup. b) The example of the revised HGT profile. Supplementary Fig. S7. The performance of GFICLEE by phylogenetic profile compares with the revised phylogenetic profile in three databases. Supplementary Fig. S8. The performance of GFICLEE compares with CLIME by the phylogenetic profi [file 12864_2021_8070_MOESM1_ESM.pdf]

GFICLEE: ultrafast tree-based phylogenetic profile method inferring gene function at the genomic-wide level

Yang Fang[1], Menglong Li[2], Xufeng Li[1], Yi Yang[1,] *

[1]Key Laboratory of Bio-Resources and Eco-Environment of Ministry of Education, College of Life Sciences, Sichuan University, Chengdu, PR China

[2]College of Chemistry, Sichuan University, Chengdu, PR China

Supplementary Figures

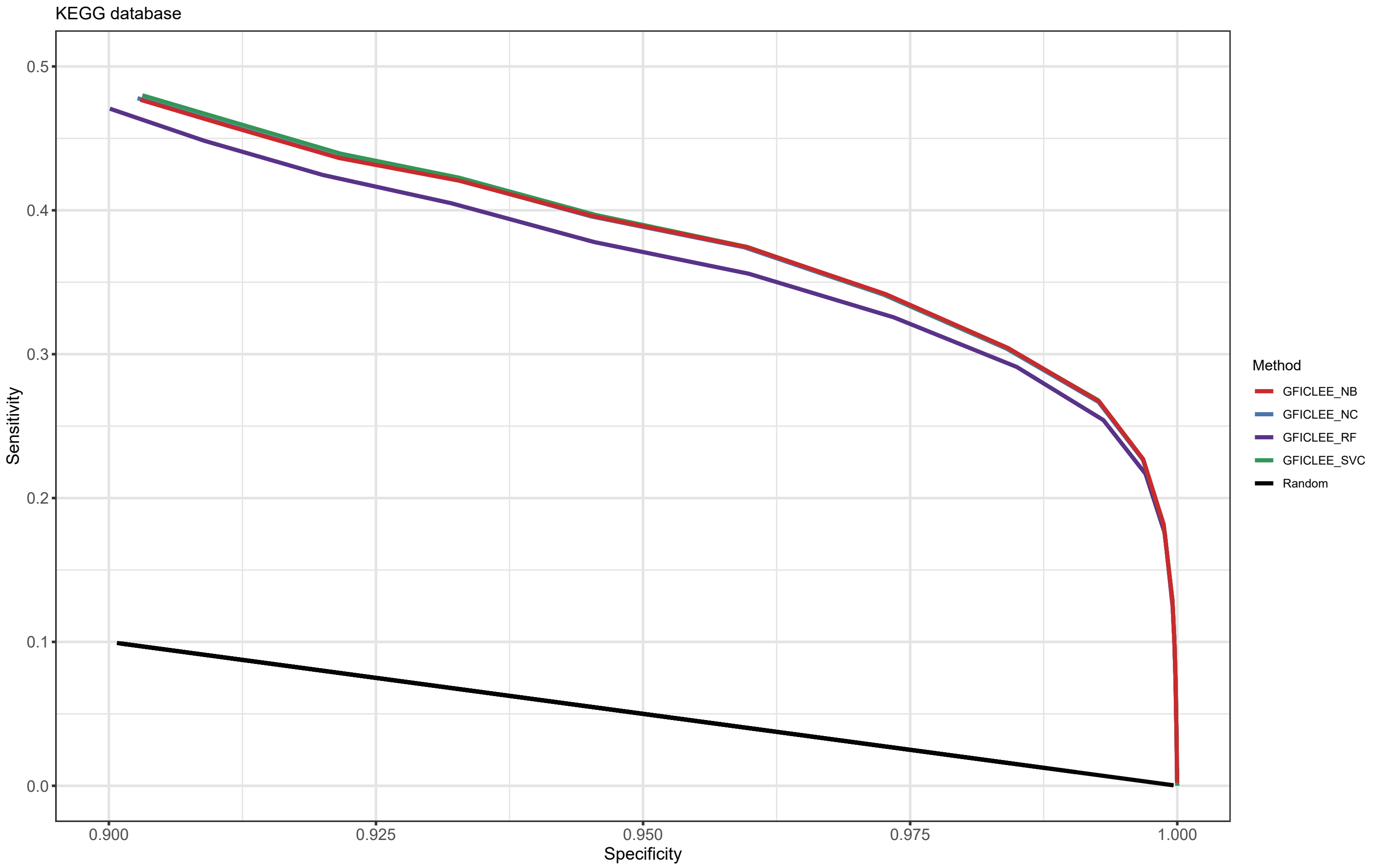

Supplementary Figure S1. The performance of different classifies methods. The performance of naive Bayesian method compares with Random Forest, Support Vector Machines and Nearest Neighbors classify methods. In the figure, NB: naive Bayesian, SVC: Support Vector Machines classification, NC: Nearest Neighbors classification and RF: Random Forest classification.

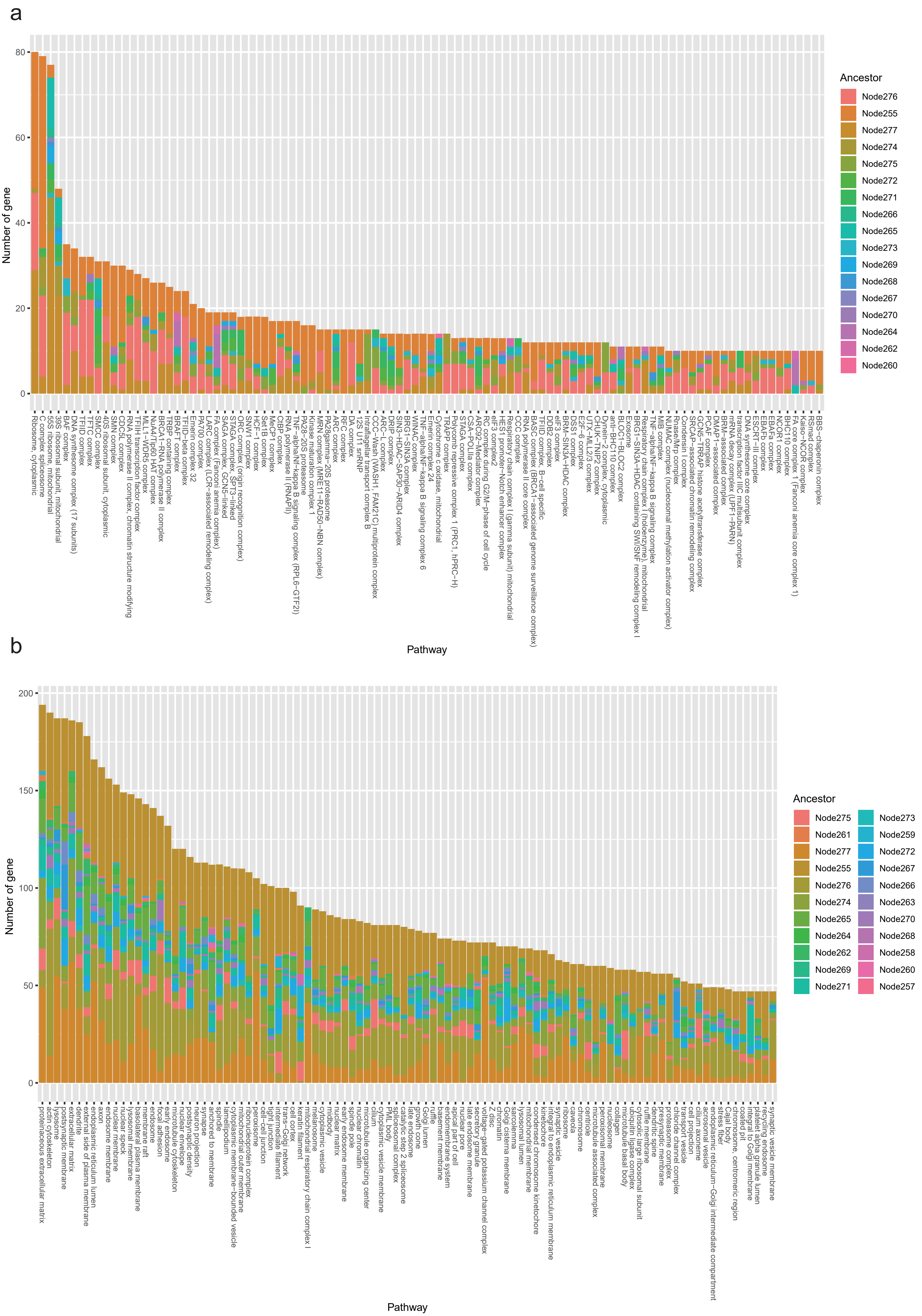
a
Number of gene
80
60
40
20
0
Ancestor
Node276
Node255
Node277
Node274
Node275
Node272
Node271
Node266
Node265
Node273
Node269
Node268
Node267
Node270
Node264
Node262
Node260
Ribosome, cytoplasmic
C complex spliceosome
55S ribosome, mitochondrial
39S ribosomal subunit, mitochondrial
BAF complex
DNA synthesome complex (17 subunits)
TFIID complex
TFTC complex
SMCC complex
40S ribosomal subunit, cytoplasmic
SMN complex
CDC5L complex
RNA polymerase II complex, chromatin structure modifying
TFIIH transcription factor complex
MLL1−WDR5 complex
NuA4/Tip60 HAT complex
BRCA1−RNA polymerase II complex
TRBP containing complex
BRAFT complex
TFIID−beta complex
Emerin complex 32
PA700 complex
LARC complex (LCR−associated remodeling complex)
FA complex (Fanconi anemia complex)
SAGA complex, GCN5−linked
STAGA complex, SPT3−linked
ORC complex (origin recognition complex)
SNW1 complex
HCF−1 complex
Set1B complex
MeCP1 complex
CtBP complex
RNA polymerase I (RNAPI)
TNF−alpha/NF−kappa B signaling complex (RPL6−GTF2I)
PA28−20S proteasome
Kinase maturation complex 1
PA28gamma−20S proteasome
MRN complex (MRE11−RAD50−NBN complex)
ARC complex
RFC complex
DA complex
12S U11 snRNP
Intraflagellar transport complex B
CCC−Wash (WASH1, FAM21C) multiprotein complex
ARC−L complex
DRIP complex
SIN3−HDAC−SAP30−ARID4 complex
BRG1−SIN3A complex
WINAC complex
TNF−alpha/NF−kappa B signaling complex 6
Emerin complex 24
Cytochrome c oxidase, mitochondrial
TRAPP complex
Polycomb repressive complex 1 (PRC1, hPRC−H)
STAGA complex
CSA−POLIIa complex
ARC92−Mediator complex
RC complex during G2/M−phase of cell cycle
eIF3 complex2
HES1 promoter−Notch enhancer complex
Respiratory chain complex I (gamma subunit) mitochondrial
CCC complex
RNA polymerase II core complex
BASC complex (BRCA1−associated genome surveillance complex)
TFIID complex, B−cell specific
DBIRD complex
eIF3 complex
BRM−SIN3A−HDAC complex
DSS1 complex
E2F−6 complex
UTX−MLL2/3 complex
CHUK−TNIP2 complex
Dynein−2 complex, cytoplasmic
anti−BHC110 complex
BLOC1−BLOC2 complex
Exosome
Respiratory chain complex I (holoenzyme), mitochondrial
BRG1−SIN3A−HDAC containing SWI/SNF remodeling complex I
TNF−alpha/NF−kappa B signaling complex
MIB complex
NUMAC complex (nucleosomal methylation activator complex)
Rnase/Mrp complex
Condensin I complex
SRCAP−associated chromatin remodeling complex
GCN5−TRRAP histone acetyltransferase complex
PCAF complex
DMAP1−associated complex
BRM−associated complex
mRNA decay complex (UPF1−PARN)
transcription factor IIIC multisubunit complex
DNA synthesome core complex
ESCRT−III complex
EBAFb complex
EBAFa complex
NCOR1 complex
BHC110 complex
FA core complex 1 (Fanconi anemia core complex 1)
Kaiso−NCOR complex
RSmad complex
BBS−chaperonin complex
Pathway
b
Number of gene
200
150
100
50
0
Ancestor
Node275
Node261
Node277
Node255
Node276
Node274
Node265
Node264
Node262
Node269
Node271
Node273
Node259
Node272
Node267
Node266
Node263
Node270
Node268
Node258
Node260
Node257
proteinaceous extracellular matrix
actin cytoskeleton
lysosome
postsynaptic membrane
extracellular matrix
dendrite
external side of plasma membrane
endoplasmic reticulum lumen
axon
endosome membrane
nuclear membrane
nuclear speck
lysosomal membrane
basolateral plasma membrane
membrane raft
endosome
focal adhesion
early endosome
microtubule cytoskeleton
nuclear envelope
neuron projection
postsynaptic density
synapse
anchored to membrane
spindle
lamellipodium
cytoplasmic membrane−bounded vesicle
mitochondrial outer membrane
ribonucleoprotein complex
peroxisome
cell−cell junction
tight junction
intermediate filament
trans−Golgi network
cell cortex
keratin filament
mitochondrial respiratory chain complex I
melanosome
cytoplasmic vesicle
midbody
nuclear matrix
early endosome membrane
spindle pole
nuclear chromatin
microtubule organizing center
cilium
cytoplasmic vesicle membrane
PML body
spliceosomal complex
catalytic step 2 spliceosome
late endosome
growth cone
Golgi lumen
ruffle
basement membrane
endomembrane system
apical part of cell
nuclear pore
late endosome membrane
secretory granule
voltage−gated potassium channel complex
Z disc
chromatin
Golgi cisterna membrane
sarcolemma
lysosomal lumen
mitochondrial membrane
condensed chromosome kinetochore
kinetochore
integral to endoplasmic reticulum membrane
synaptic vesicle
ribosome
caveola
chromosome
centriole
microtubule associated complex
peroxisomal membrane
nucleosome
collagen
microtubule basal body
cytosolic large ribosomal subunit
ubiquitin ligase complex
ruffle membrane
dendritic spine
presynaptic membrane
proteasome complex
chloride channel complex
transport vesicle
cell projection
cilium axoneme
acrosomal vesicle
endoplasmic reticulum−Golgi intermediate compartment
stress fiber
Cajal body
chromosome, centromeric region
coated pit
integral to Golgi membrane
platelet alpha granule lumen
recycling endosome
synaptic vesicle membrane
Pathway

c

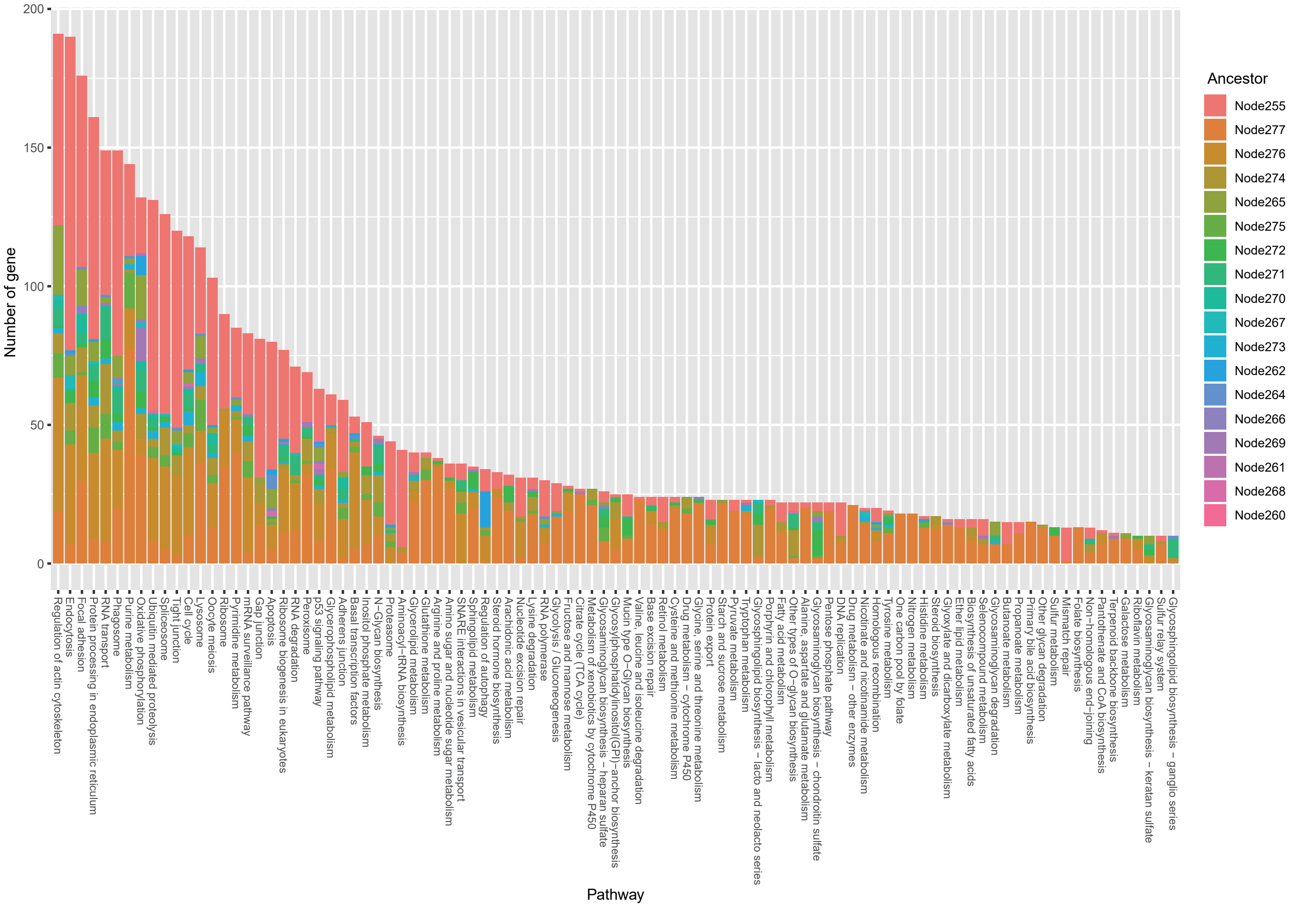

Supplementary Figure S2. The common ancestor of genes in different databases. a) The common ancestor of genes in the human pathway with CORUM database. b) The common ancestor of genes in the human pathway with GO database. c) The common ancestor of genes in the human pathway with KEGG database.

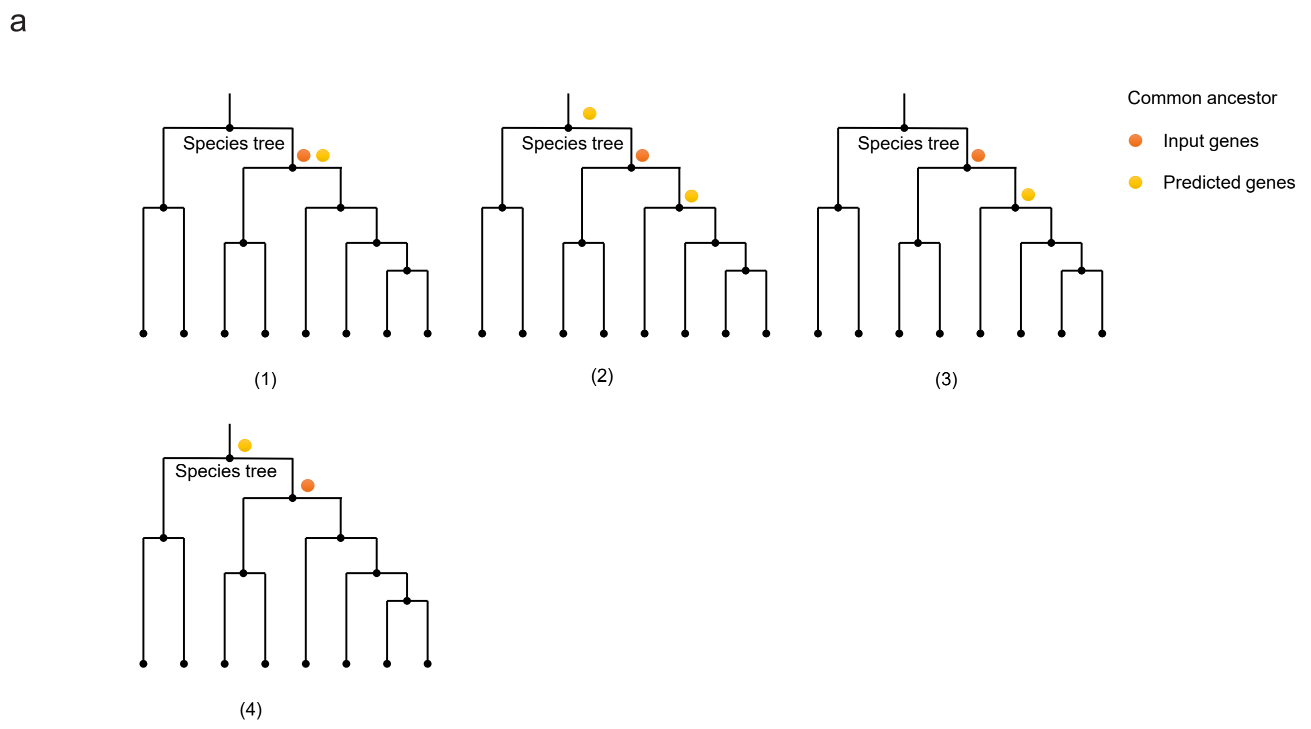

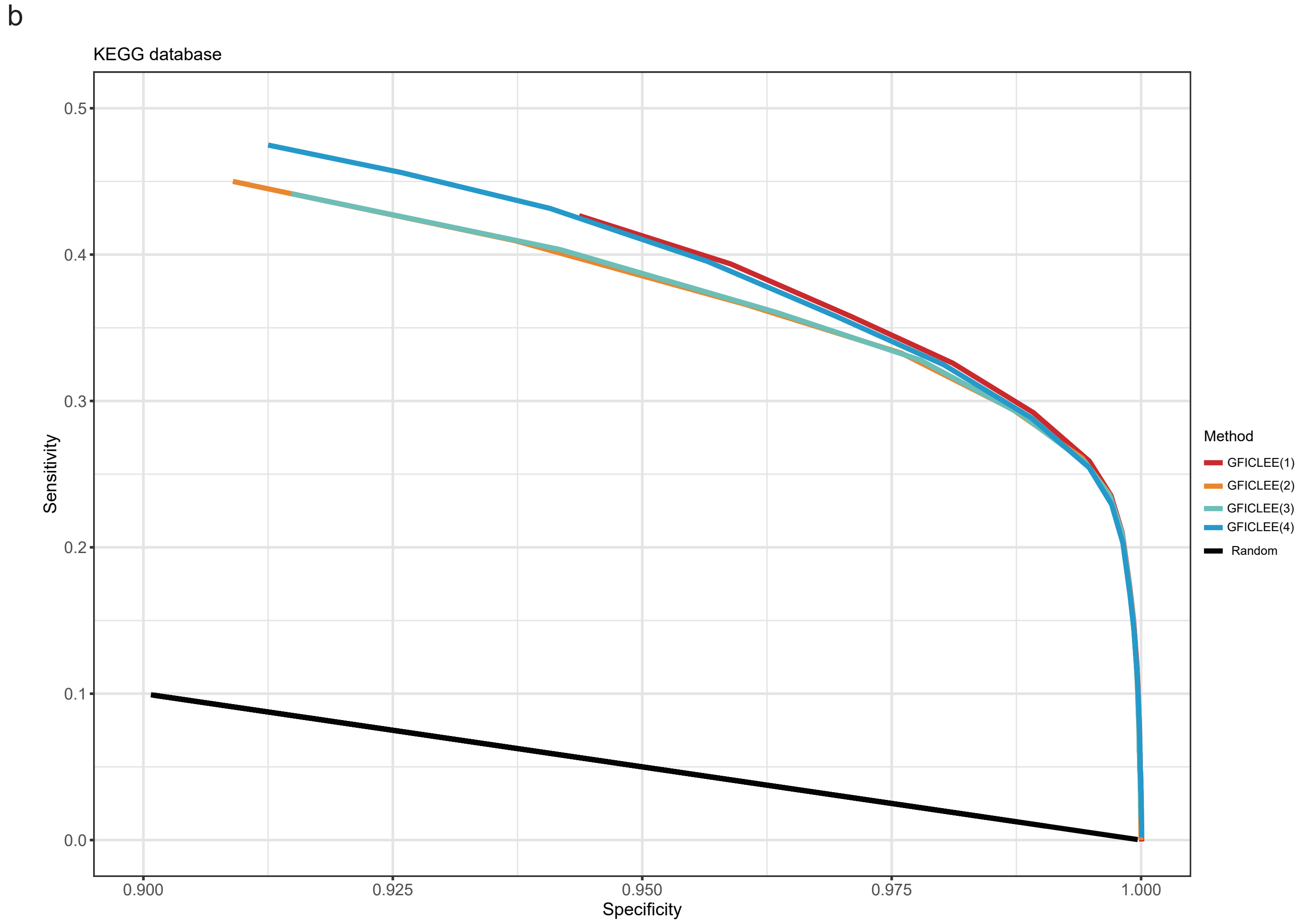

Supplementary Figure S3. The comparison of genes functions appeared in later or earlier than input genes. a) Setting the search node is same with input genes node (1), Setting the search node later and earlier than input gene node (2), Setting the search node later than input genes node (3), Setting the search node earlier than input genes node (4). b) The performance of setting the different search gain node.

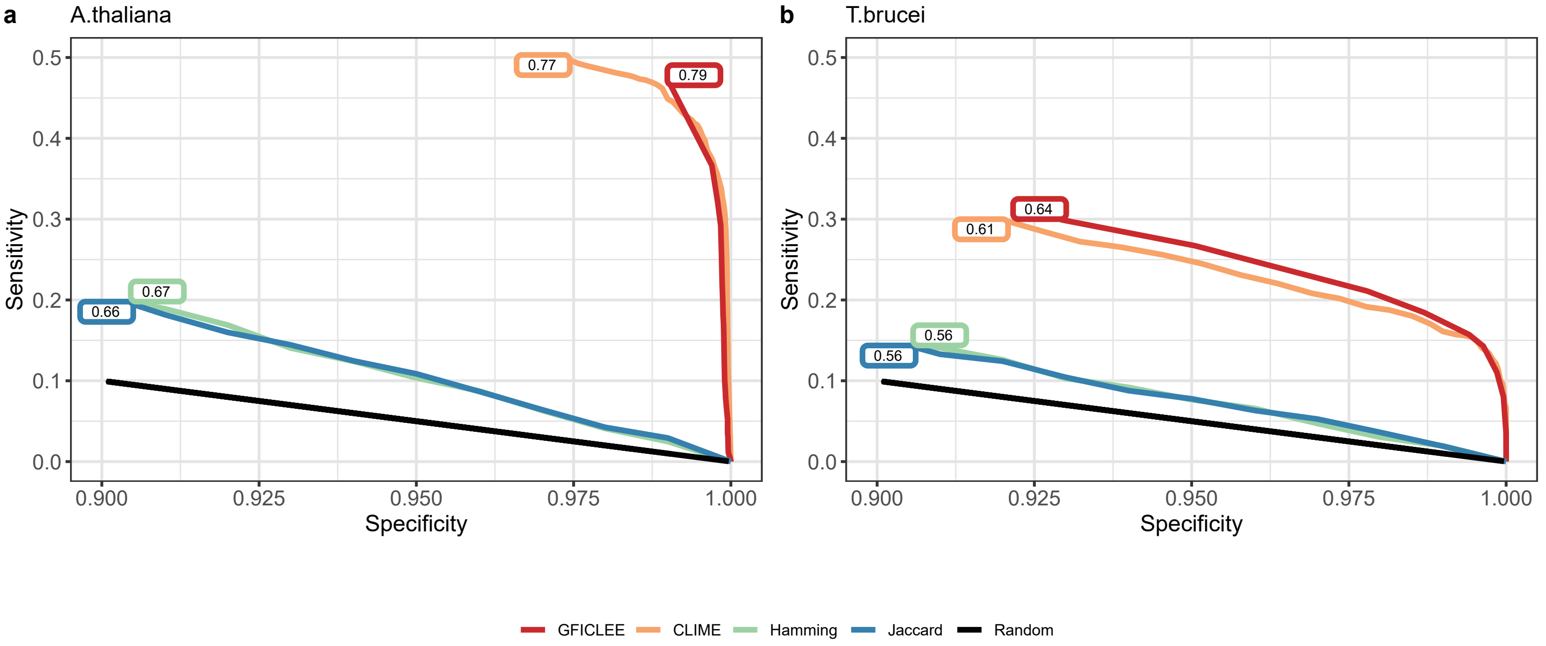

Supplementary Figure S4. The performance of GFICLEE compares with existing approaches by different genomes. a) The *A. thaliana* metabolic and signaling pathways. b) The *T. brucei* metabolic and signaling pathways.

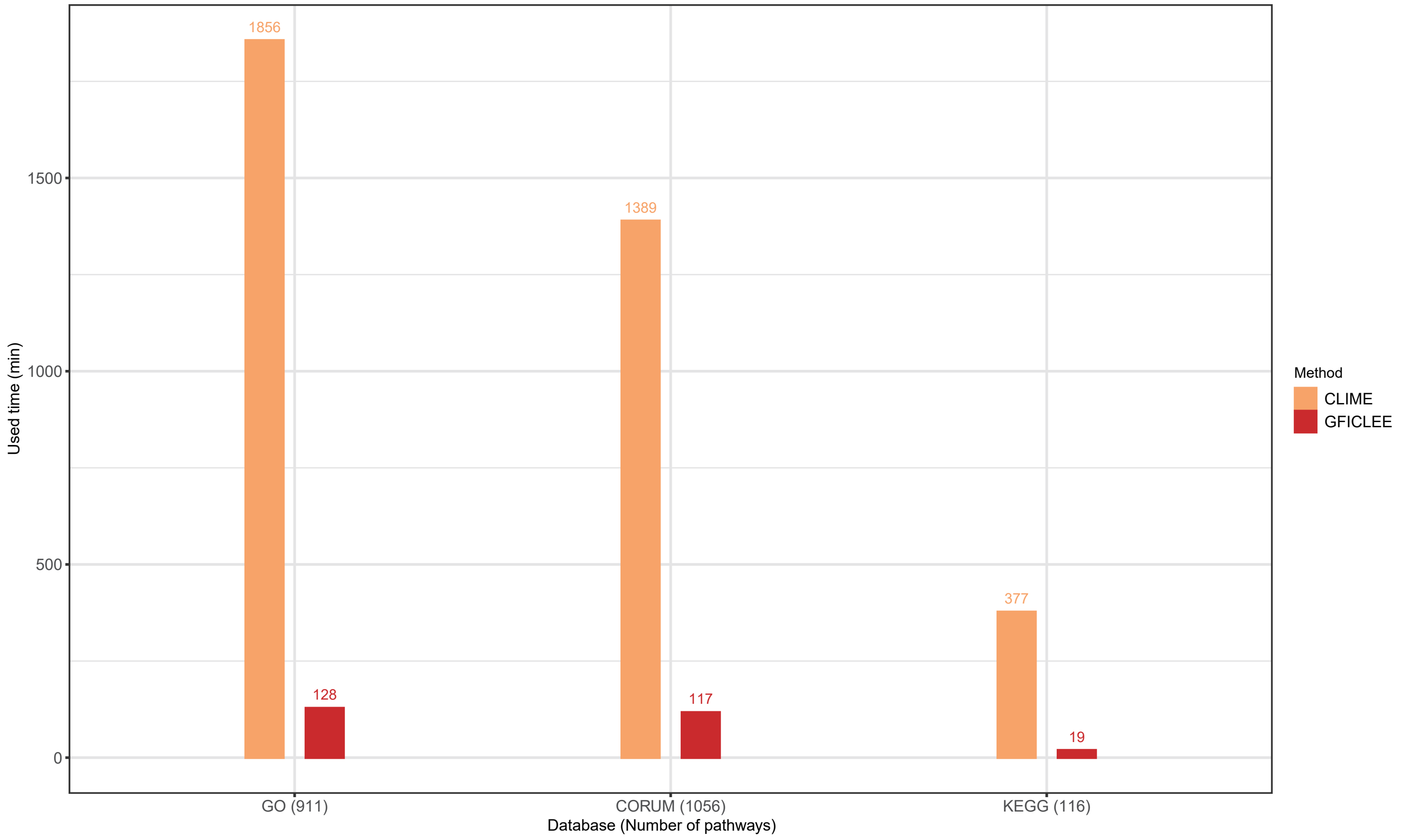

Supplementary Figure S5. The computational time of three different databases. The test used the human genome with the GO database that contains 911 pathways, CORUM contains 1056 pathways and KEGG with 116 pathways. The test parallel running with 10 cores for CLIME and GFILEE software, respectively.

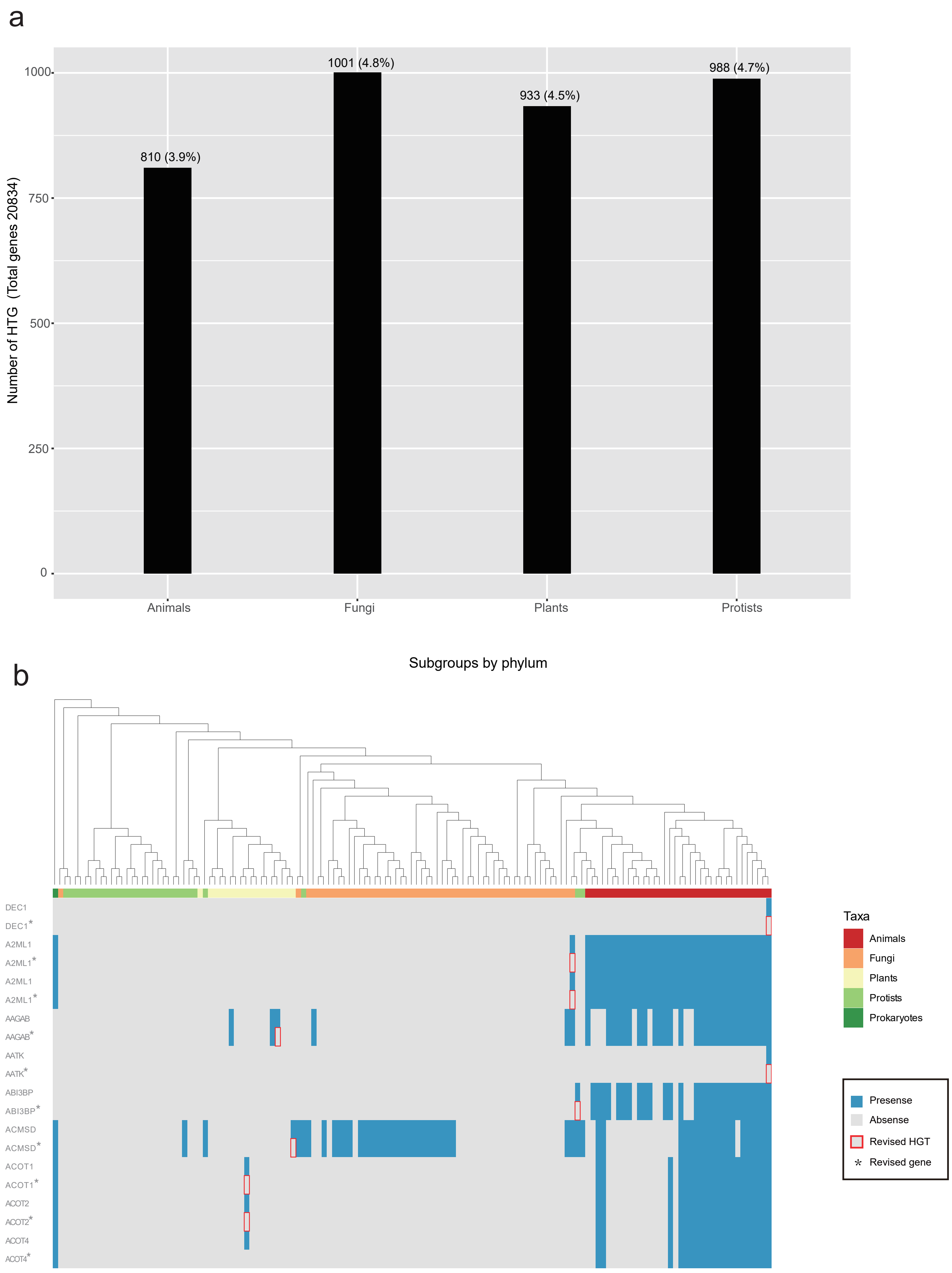

Supplementary Figure S6. The HGT events occurred in the human phylogenetic profile. a) The HGT events occurred in each subgroup. b) The example of the revised HGT profile.

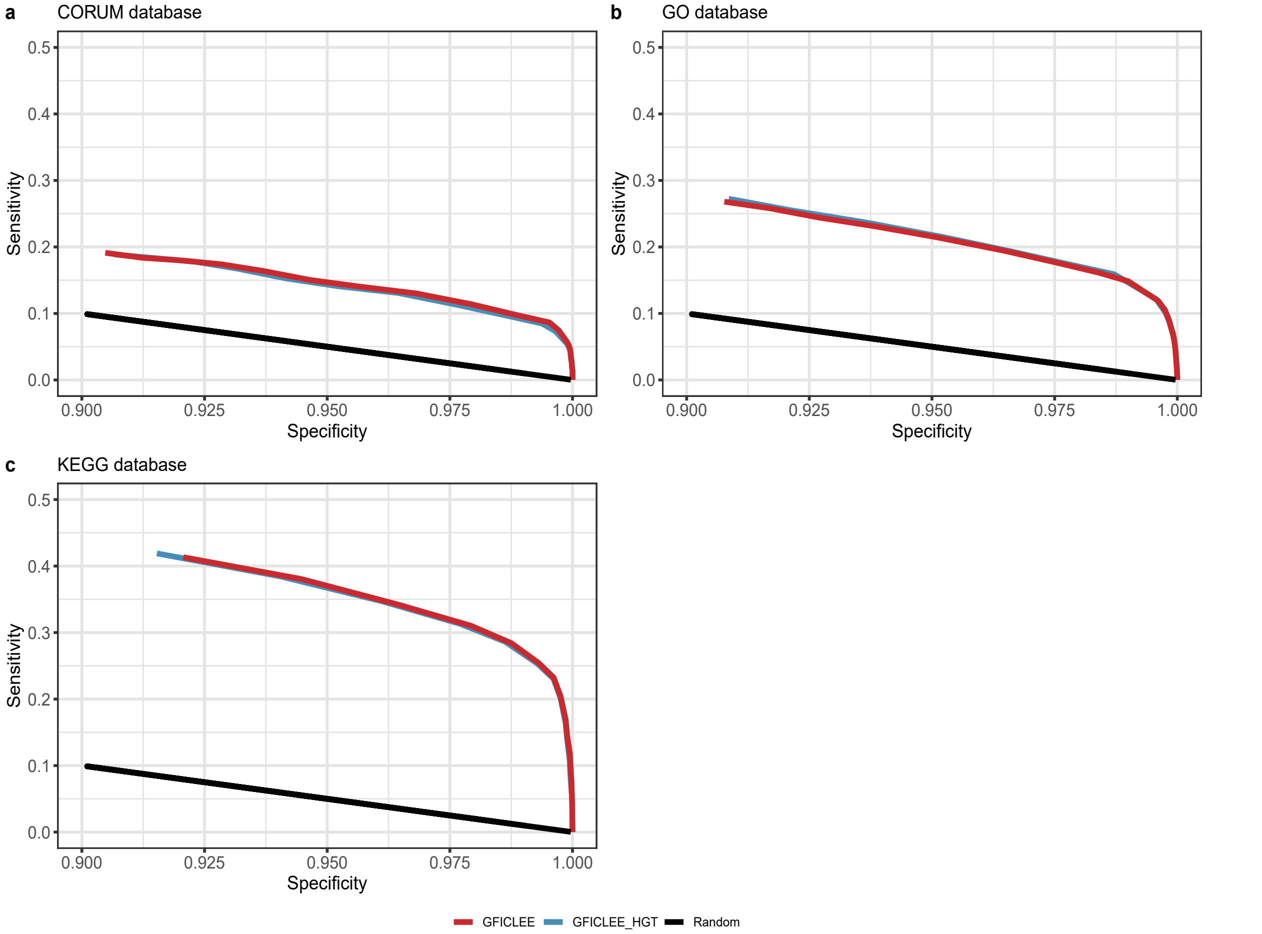

Supplementary Figure S7. The performance of GFICLEE by phylogenetic profile compares with the revised phylogenetic profile in three databases.

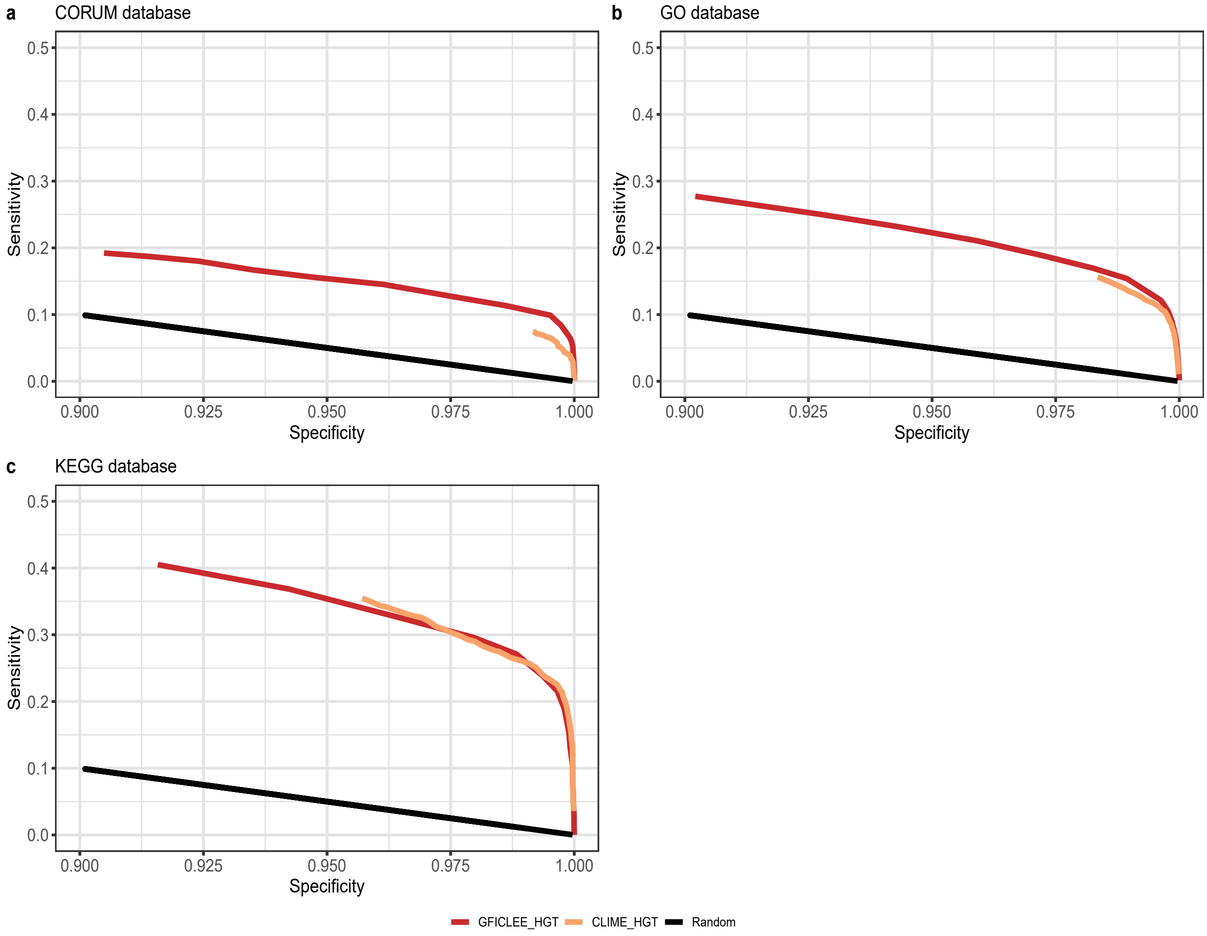

Supplementary Figure S8. The performance of GFICLEE compares with CLIME by the phylogenetic profile that removes suspected HGT genes.

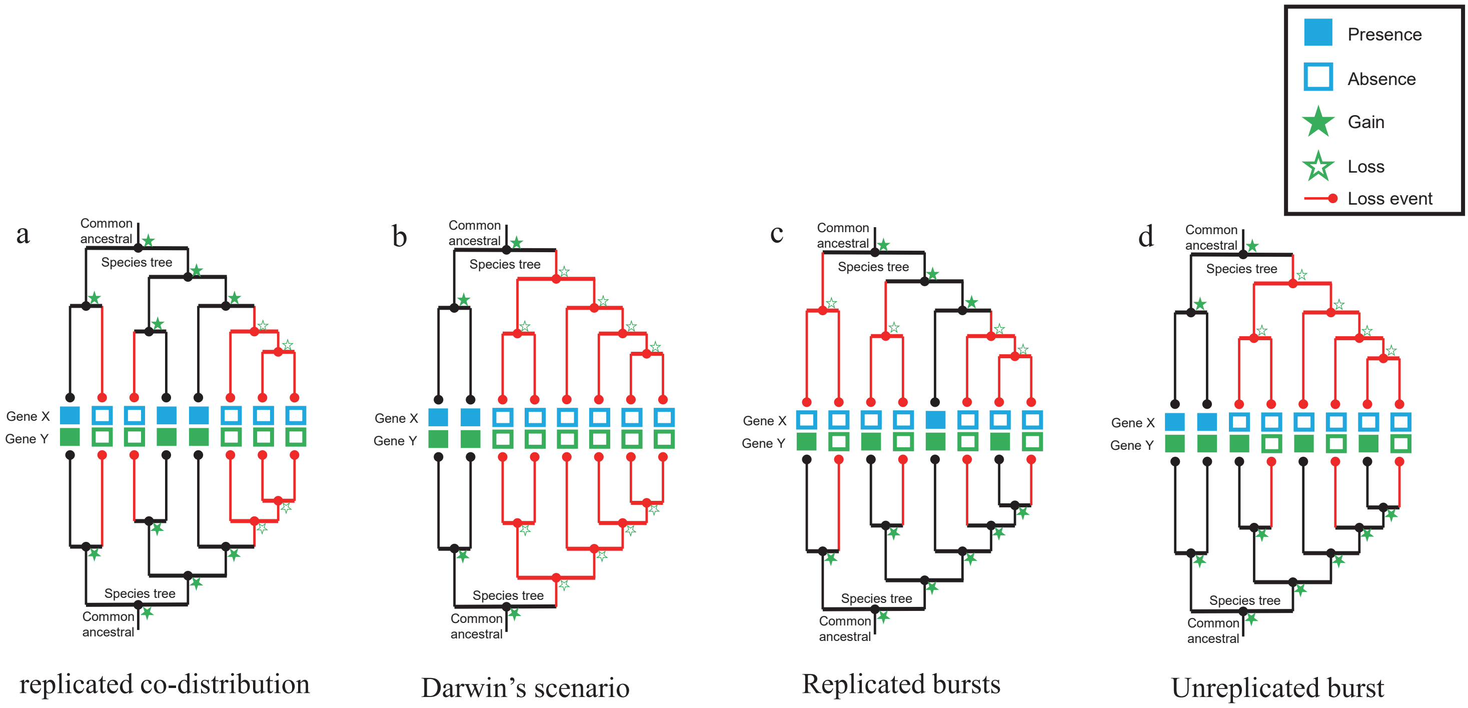

Supplementary Figure S9. GFICLEE algorithm corresponding four scenarios for the evolution of states of characters gene X and Y. a) The replicated co-distributions contain single and continuous loss events. b) Darwin's scenario only contains continuous loss events. c) and d) The scenarios of Replicated bursts and Unreplicated burst, respectively.

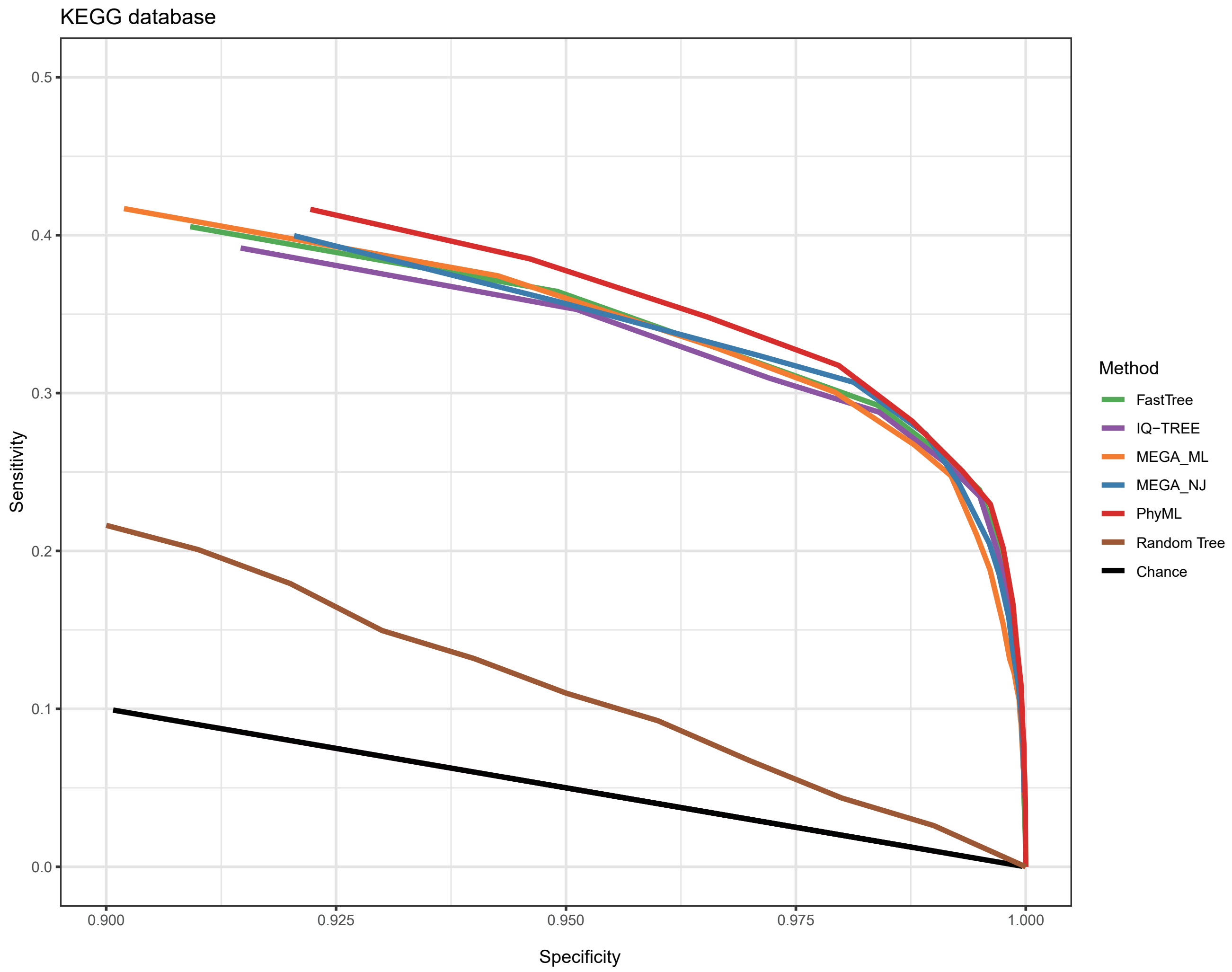

Supplementary Figure S10. The performance of GFICLEE with different software reconstruct species tree and random topology tree. FastTree, IQ-TREE, MEGA_ML and PhyML are maximum likelihood method reconstruct species tree. The MEGA_ML is the Neighbor-Joining method reconstruct species tree.

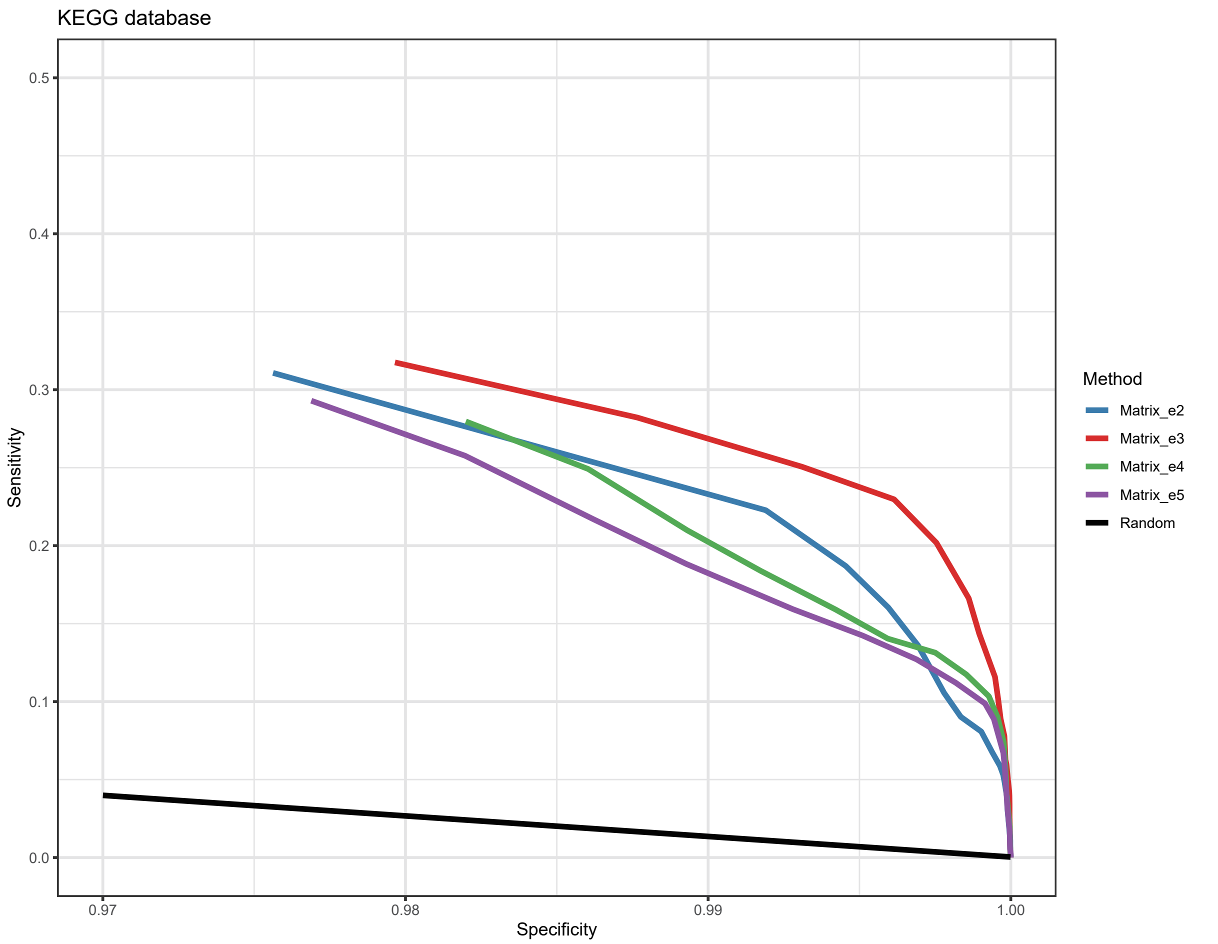

Supplementary Figure S11. The performance of GFICLEE with different threshold generate phylogenetic matrix. The various BLASTP threshold to (E-value <10-2, E-value <10-3, E-value <10-4 and E-value <10-5) generate ortholog matrix and test the performance of GFICLEE.

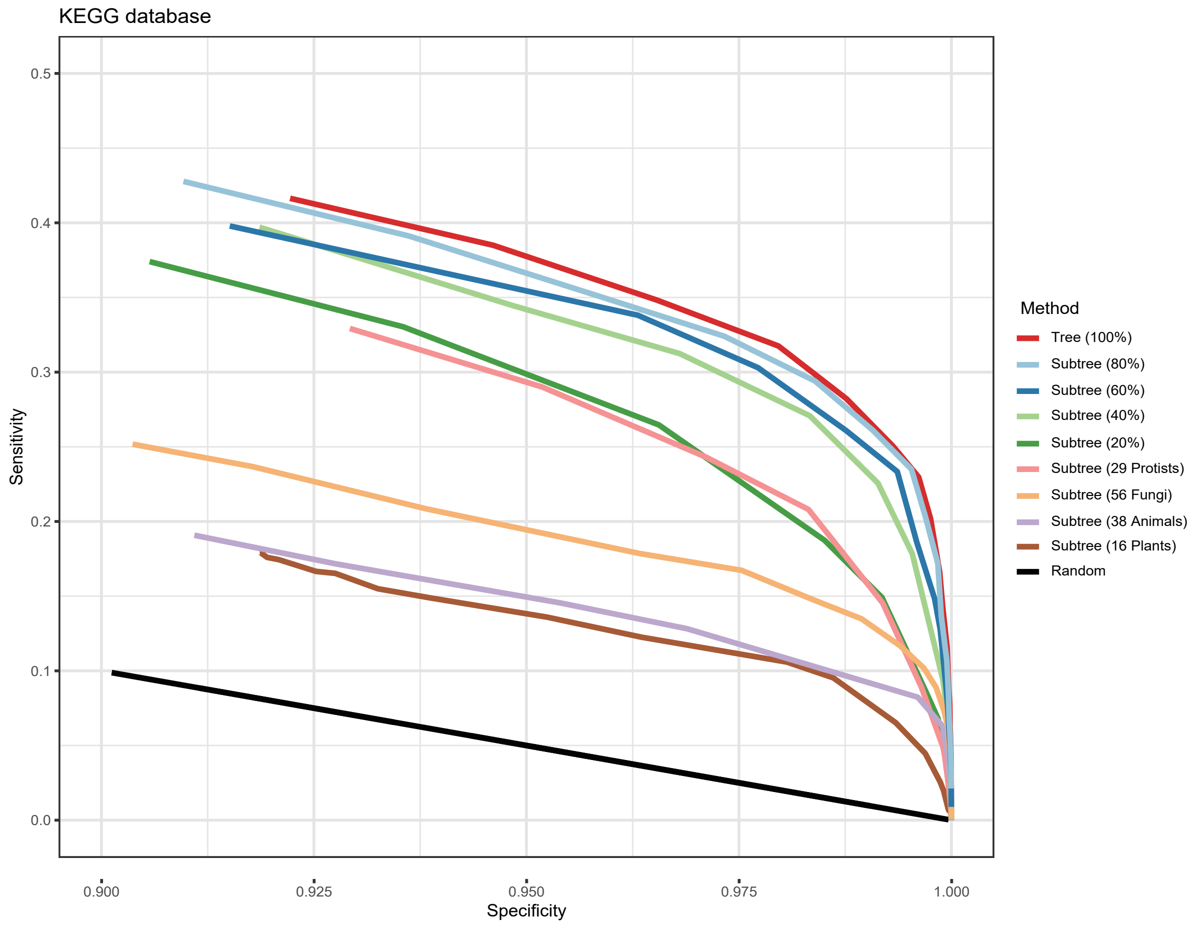

Supplementary Figure S12. The taxon sampling effect on the performance of GFICLEE. We extract species from each phylum classification at a ratio of 20%, 40%, 60%, and 80% to generate subtrees. The contains each phylum only also extracted for the performance test.
